# The cost structure of routine infant immunization services: a systematic analysis of six countries

**DOI:** 10.1093/heapol/czx067

**Published:** 2017-05-31

**Authors:** Fangli Geng, Christian Suharlim, Logan Brenzel, Stephen C Resch, Nicolas A Menzies

**Affiliations:** 1 Center for Health Decision Science, Harvard T. H. Chan School of Public Health, Boston, MA, USA,; 2 Bill & Melinda Gates Foundation, Seattle, Washington, DC, USA and; 3 Department of Global Health and Population, Harvard T. H. Chan School of Public Health, Boston, MA, USA

**Keywords:** Health care costs, delivery of healthcare, cost structure, resource allocation, immunization

## Abstract

Little information exists on the cost structure of routine infant immunization services in low- and middle-income settings. Using a unique dataset of routine infant immunization costs from six countries, we estimated how costs were distributed across budget categories and programmatic activities, and investigated how the cost structure of immunization sites varied by country and site characteristics. The EPIC study collected data on routine infant immunization costs from 319 sites in Benin, Ghana, Honduras, Moldova, Uganda, Zambia, using a standardized approach. For each country, we estimated the economic costs of infant immunization by administrative level, budget category, and programmatic activity from a programme perspective. We used regression models to describe how costs within each category were related to site operating characteristics and efficiency level. Site-level costs (incl. vaccines) represented 77–93% of national routine infant immunization costs. Labour and vaccine costs comprised 14–69% and 13–69% of site-level cost, respectively. The majority of site-level resources were devoted to service provision (facility-based or outreach), comprising 48–78% of site-level costs across the six countries. Based on the regression analyses, sites with the highest service volume had a greater proportion of costs devoted to vaccines, with vaccine costs per dose relatively unaffected by service volume but non-vaccine costs substantially lower with higher service volume. Across all countries, more efficient sites (compared with sites with similar characteristics) had a lower cost share devoted to labour. The cost structure of immunization services varied substantially between countries and across sites within each country, and was related to site characteristics. The substantial variation observed in this sample suggests differences in operating model for otherwise similar sites, and further understanding of these differences could reveal approaches to improve efficiency and performance of immunization sites.

## Introduction

Key Messages

- Site-level costs (incl. vaccines) represented 77–93% of national routine infant immunization costs across six countries.
- Labour and vaccine costs comprised 14–69% and 13–69% of site-level cost, respectively across six countries.
- 48–78% of site-level costs were devoted to service provision (facility-based or outreach) across six countries.
- Across all countries, more efficient sites (compared with sites with similar characteristics) had a lower fraction of costs devoted to labour.

Vaccination is one of the most cost-effective approaches for preventing infectious disease and improving health in countries affected by vaccine-preventable diseases (Ozawa *et al.* 2016). Since 2000, the Global Alliance for Vaccine and Immunization (GAVI) has supported high burden countries to introduce new vaccines and expand vaccination coverage (Henderson *et al.* 2016). GAVI’s financial support is tied to country income, and as a county’s per-capita income increases so do their co-financing responsibilities, until they eventually become fully self-financing. As countries look to replace funding previously supported by GAVI, efficiency and sustainability are critical programme concerns, in addition to maximizing population coverage and increasing the range of disease against which children are protected (Portnoy *et al.* 2015; Kallenberg *et al.* 2016).

Improving efficiency and sustainability is difficult without understanding how funds are currently spent. To strengthen the evidence base around immunization service costs, a multi-country study on routine immunization costing and financing (EPIC) was conducted in Benin, Ghana, Honduras, Moldova, Uganda and Zambia (Brenzel *et al.* 2015). Detailed data on resource use and programme performance were collected from >300 immunization sites using a standardized approach (Brenzel, 2014), and summary information for each country have been reported (Goguadze *et al.* 2015; Guthrie *et al.* 2015; Janusz *et al.* 2015; Le Gargasson *et al.* 2015; Schutte *et al.* 2015). These analyses demonstrated substantial variation in total and unit costs for immunization services within and between countries, with higher unit costs associated with country per-capita income, and countries supporting a greater share of total costs compared with earlier estimates (Brenzel *et al.* 2015).

In some cases these EPIC country studies reported the distribution of costs across budget categories, describing differences in cost distributions by urban/rural status (Schutte *et al.* 2015), facility type (Goguadze *et al.* 2015) and service volume (Goguadze *et al.* 2015). These results reinforce earlier research showing substantial variation between sites: in a study in Peru conducted in 2002, Walker *et al.* (2004) revealed large variation across all cost categories between different types of facilities, with the proportion of costs devoted to personnel varying from 15 to 40%. Similarly, Robertson *et al.* (1984) reported inter-site variation in the cost share of immunization devoted to vaccines ranging from 13 to 30% in Gambia. Given this evidence, we undertook a reexamination of the EPIC data to systematically describe how costs differed across budget categories and programmatic activities between countries, and between sites within each country.

Systematic cost structure analysis has been employed by a range of organizations and utilities to monitor spending and improve resource allocation (Dinusha and Jaai 2012; Vans and Walker 1972; Jacobs 1993; Gagné 1990). However, use of these methods within publicly funded health programmes is not routine. Although public health programmes face different goals and incentives to private companies, financial sustainability and efficiency are common concerns, particularly when budgets are highly constrained. Understanding how cost structure varies between service outlets can identify opportunities to reduce waste, or reveal efficient operating models. Cost structure analysis can also support programme budgeting exercises particularly when the resources available for each part of the budget may not be completely fungible. Finally, understanding the cost distribution for different types of sites provides information about how resource requirements for different parts of the budget may change as programmes mature and vaccine schedules change. By undertaking systematic cost structure analysis for each of the six EPIC countries, this analysis is intended to extend the insights of earlier analyses and provide a fine-grained understanding of the distribution of immunization programme costs.

## Methods

### Overview

We based our analysis on a unique dataset describing resource use and programme outputs for 316 immunization sites, collected through the EPIC studies (Center for Health Decision Science 2015). We categorized cost data according to the budget category and the programmatic activity to which resources were devoted. The analysis is composed of three parts: (1) a description of the cost shares of immunization for each of the six EPIC countries, (2) regression analyses to determine how the cost shares of site-level immunization are related to observable site characteristics (such as service delivery volume and rurality), and (3) regression analyses to investigate how the cost shares of site-level immunization are related to an index describing the relative efficiency of each site compared with sites with similar characteristics.

### Data collection and management

The EPIC studies collected data on routine immunization costs in Benin, Ghana, Honduras, Moldova, Uganda and Zambia (Goguadze *et al.* 2015; Guthrie *et al.* 2015; Janusz *et al.* 2015; Le Gargasson *et al.* 2015; Schutte *et al.* 2015). Background information on income level, infant population, vaccine coverage and immunization schedule for each country is given in Supplementary Tables S1 and S2. In each country a representative sample of sites was selected as a multi-stage cluster sample, and sampling weights calculated as the inverse of the selection probability (Brenzel 2014). These weights were used in analyses to reweight the sample to be nationally representative for each country. Data were collected using a standardized approach and describe resource utilization and programme outputs for each sampled site (319 total sites). These data describe infant immunization activities conducted during January–December 2011. The costing adopted a provider perspective, including site-level costs incurred by all organizations involved in supporting immunization services, and excluding costs incurred by programme clients. Data on resource utilization, input prices, service volume and other site characteristics were cleaned and organized into a single dataset (Center for Health Decision Sciences 2015). For this analysis, four sites were excluded due to missing data or where key variables could not be verified, resulting in an analytic sample size of 315 (44, 50, 71, 50, 49 and 51 for Benin, Ghana, Honduras, Moldova, Uganda and Zambia, respectively). The economic costs of routine infant immunization services (0–12 months of age) were estimated retrospectively, and categorized according to the budget category of each cost input and programmatic activity to which resources were devoted (Table 1). For resources supporting infant immunization as well as services for older ages, we allocated costs proportional to doses delivered. Categorizations were based on definitions adopted by the original county studies, with small cost categories collapsed for clarity. Categories were defined to be mutually exclusive and exhaustive, such that the sum across each set of categories was equal to the total cost for each site. Costs are reported as 2011 US dollars.

**Table 1.** Cost categorization

Categorization	Category name	Details
Budget category	Labour	Shared and immunization-specific personnel salary and volunteer labour estimated as the market value.
	Vaccine	Vaccines, including wastage and supplies, including syringes, diluent, safety boxes and other supplies used for administration of vaccines.
	Transport	Value of all the vehicles and modes of transport, maintaining vehicles and other transport for immunization-related activities and other immunization-related transport, including both facility-based and outreach services.
	Cold chain	All cold chain equipment used to store and transport vaccines, related energy cost and the cost of ice.
	Infrastructure	Building areas, utilities and communication, costs related to building overheads, other equipment, such as computers, printers, furniture, other medical equipment used for immunization-related activities and printing costs,related to immunization-related materials.
	Per diem	Any allowances paid to paid or volunteer workers for immunization-related activities.
Programmatic activity	Facility-based services	Time and resources spent on the act of administering the vaccine to children within the facility/compound and costs of vaccines delivered through facilities.
	Surveillance	Disease surveillance, following-up post-vaccination events and active cases of diseases that were prevented by vaccination, record keeping, HMIS, monitoring and evaluation.
	Programme management	Programme management, training and supervision.
	Outreach services	Time and resources spent for outreach and costs of vaccines delivered through outreach.
	Social mobilization	Time and resources spent mobilizing the community and households, and advocating for vaccination.
	Supply chain	Cold chain equipment used to store and transport vaccines, cold chain energy cost, the cost of ice, and time and resources spent on vaccine collection, distribution and storage.

### Cost shares by country

Above-site level costs were extracted from EPIC country reports and categorized by budget category (Agence de Medecine Preventive 2014; Gotsadze *et al.* 2014; Guthrie 2016; Janusz *et al.* 2014; Le Gargasson *et al.* 2014; Schütte *et al.* 2014). We attributed all vaccine costs to the site level, to demonstrate how these change as a fraction of site-level costs, and allow for differences in wastage rates as a function of site characteristics. For each country, we estimated the average site-level cost shares across budget categories and programme activities. To do so we calculated the average site-level cost for each category, using reported survey weights, and divided by the average total site-level cost.

### Relationship between site characteristics and cost shares

We used regression analysis to investigate systematic relationships between observable site characteristics and the site-level cost shares. Characteristics included facility type [hospital/non-hospital], area type [rural/non-rural], facility ownership [government/non-government], distance [between the facility and the vaccine distribution centre] and service delivery volume [total doses delivered]. Facility type, area type, facility ownership and distance to the vaccine distribution centre all describe fixed features of a site’s operating environment that could lead to differences in cost structure. Service delivery volume is a major cost determinant, with several earlier studies describing economies of scale for total costs (Ahanhanzo *et al.* 2015; Maceira *et al.* 2015). We included service volume in these regressions to describe how the cost shares change as a function of service volume. Table 2 presents descriptive statistics for variables used in regression analyses.

**Table 2.** Descriptive statistics for all variables in the sample for each country^a^

Variables	Benin	Ghana	Honduras	Moldova	Uganda	Zambia
Sample size	44	50	71	50	49	51
Total doses	6791 (4490)	3512 (3775)	4244 (7175)	557 (1172)	6561(12144)	7069 (11343)
Area type (Rural/All)	25/45	31/50	53/71	42/50	29/49	36/51
Ownership (Govt owned/All)	40/44	47/50	71/71	50/50	37/49	49/51
Facility type (Hospital/All)	0/45	6/50	3/71	0/50	13/49	4/51
Distance to vaccine collection point (km)	14.6 (17.8)	7.5 (11.3)		19.6 (13.1)	12.9 (12.6)	50.2 (44.8)
Costs of budget category (USD)						
Labour	2924 (2284)	12 235 (8883)	13 138 (17 423)	5289 (10 016)	4553 (4471)	14 815 (8937)
Vaccine	14 094 (9515)	6322 (6709)	15 675 (23 591)	1006 (2190)	7504 (14 617)	9050 (13904)
Transport	660 (697)	1177 (2686)	105 (363)	76 (92)	1960 (3202)	1967 (2668)
Cold chain	1876 (967)	266 (272)	389 (309)	58 (21)	666 (1080)	491 (510)
Infrastructure	576 (551)	320 (1179)	535 (705)	1102 (2113)	676 (805)	1413 (851)
Per diem	89 (128)	58 (236)	535 (870)	6 (16)	159 (182)	2668 (3311)
Costs of programmatic activity (USD)						
Facility-based services	12 662 (8085)	5450 (7398)	18 625 (27326)	3485 (6376)	6643 (9390)	10 383 (13 434)
Surveillance	966 (758)	4259 (4011)	3731 (6985)	1072 (2228)	559 (584)	1428 (1506)
Programme management	548 (787)	1394 (1436)	1731 (2404)	1808 (3610)	1634 (1569)	3382 (2950)
Outreach services	3275 (4219)	5781 (5671)	2870 (4097)		4704 (7158)	9991 (7431)
Social mobilization	456 (411)	1863 (2418)	2030 (2675)	885 (1873)	246 (316)	2771 (3236)
Supply chain	2312 (1108)	1631 (2786)	1390 (1266)	286 (320)	1732 (1937)	2450 (1568)

a Sample size values represent the number of sites included in the main analysis for each country. All other values in table represent unweighted means for each county, and values in parentheses represent standard deviations.

First, we estimated cost functions for each budget category and programmatic activity, by regressing the logged costs observed for a given cost category against logged total doses (including linear and quadratic terms), area type, facility type, ownership, and distance to vaccine collection point.

$$\log\left({Cost}_{Category\;i}\right)=\;\beta_{i0}+\beta_{i1}\cdot \left(facility\;type\right)+\beta_{i2}\cdot \left(ownership\right)+\beta_{i3}\cdot \left(area\;type\right)+\beta_{i4}\cdot \left(distance\right)+\;\beta_{i5}\cdot \left(\log\left(doses\right)\right)+\;\beta_{i6}\cdot ({\log\left(doses\right)}^{2})+\varepsilon_{i}$$

For each set of categories (e.g. the six budget categories) we estimated the six regression equations simultaneously using Zellner’s seemingly unrelated regression (SUR) framework (Zellner 1962). This method allows for correlation of residuals across regression equations, and has been used previously for analyses of cost shares where residuals cannot be assumed to be independent (Binswanger 1974; Stover 1986). Regression equations were estimated separately for each country, to allow coefficient estimates and residual correlations to vary across countries.

The fitted regression equations were used to simulate two sets of outcomes:

1. ‘Change in the cost per dose for individual cost categories associated with a given change in site characteristics’*:* for each cost category, we predicted the average cost for different levels of the variable of interest, holding other variables constant at their empirical distribution in the sample (taking account of the survey weights).
2. ‘Change in the distribution of cost shares associated with a given change in site characteristics’*:* using the results obtained under (1), we divided the predicted cost for each cost category by the predicted total cost (i.e. sum of predicted average costs across all cost categories) to estimate the cost share.

Although service volume was included in regression equations as a continuous variable, we present results for quintiles of the distribution of service volume in the sample (e.g. the results for the lowest quintile show the cost shares for the smallest 20% of sites, controlling for other determinants). To calculate these quintiles we divided the distribution of service volume in each country into five equally sized groups, and evaluated results for the median value of each quintile. Measures of uncertainty (confidence intervals, *P*-values) were estimated by resampling the fitted coefficient values and variance-covariance matrices, with 10 000 replicates. Analyses were conducted in R (R Core Team 2016), and regressions were estimated using the systemfit package (Henningsen and Hamann 2007).

### Relationship between operating efficiency and cost shares

We conducted a separate regression analysis to explore how costs shares were related to site operating efficiency, controlling for other site characteristics. First, we created an index to describe the relative efficiency of each site, by regressing logged total costs against the site characteristics described in the previous section. This index was used as a relative measure of site operating efficiency (ability to achieve the same service volume as other similar sites at lower cost). This method is similar to other approaches for estimating input efficiency based on parametric cost functions, such as corrected ordinary least squares (Aigner and Chu 1968) and stochastic frontier analysis (Aigner *et al.* 1977) though no efficient frontier is estimated. Regression equations were fit separately for each country.

$$\log\left(TC\right)=\;\beta_{i0}+\beta_{i1}\cdot \left(facility\;type\right)+\beta_{i2}\cdot \left(ownership\right)+\beta_{i3}\cdot \left(area\;type\right)+\beta_{i4}\cdot \left(distance\right)+\;\beta_{i5}\cdot \left(\log\left(doses\right)\right)+\;\beta_{i6}\cdot \left({\log\left(doses\right)}^{2}\right)+\varepsilon_{i}^{\prime }$$

The residuals from this regression ($\varepsilon^{'}$) represent the log of the ratio of costs observed for each site compared with the overall trend line, with values greater than zero indicating relatively higher costs, controlling for the determinants included in the regression, and below zero indicating relatively lower costs. We used these residuals as a simple inefficiency index, divided them into quintiles and taking the median of each quintile to represent the efficiency level in that quintile. As this regression equation includes the same terms considered in the earlier regression equations, this index effectively combines all variation in costs not captured by facility type, area type, facility ownership, distance to the vaccine distribution centre and service delivery volume.

The efficiency index was used as the right-hand side variable in a set of SUR regression models similar to those described in ‘Relationship between site characteristics and cost shares’ section. We fit these cost functions for each budget category and programmatic activity, by regressing the logged costs observed for a given cost category against a set of predictors including the efficiency index (as linear and quadratic terms) and an intercept.

$$\log\left({Cost}_{Category\;i}\right)=\;\beta_{i0}+\beta_{i1}\cdot \left(efficiency\right)+\beta_{i2}\cdot ({efficiency}^{2})+\varepsilon_{i}^{\prime \prime }$$

Although the coefficient values from these equations are difficult to interpret directly, they can be used to estimate how the cost shares across budget categories and programme activities varied by efficiency level. Similar to the discretization of service volume described under ‘Relationship between site characteristics and cost shares’ section, we present results for quintiles of the distribution of the efficiency index (e.g. the results for the lowest quintile represent the cost shares for the least efficient 20% of sites, controlling for other determinants). To do so we divided the distribution of the efficiency index in each country into five equally sized groups, and evaluated results for the median value of each quintile.

## Results

### Cost shares by country

**Figure 1.**
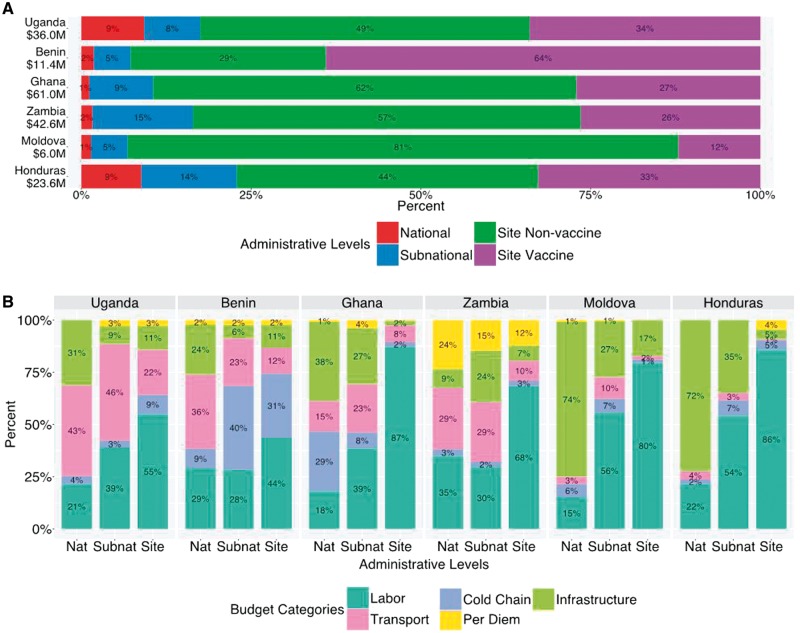
Share of total costs for infant routine immunization by administrative levels (Panel A) and share of non-vaccine service delivery costs by budget category for each administrative level (Panel B). Countries are displayed in increasing order of per capita GDP. Text shows percentage of costs in each category. Values for categories with percentage <0.5% are not shown. In panel A, the numbers under countries’ names represent the estimated total cost in each country. Some percentages don’t add up to 100%, due to rounding. In Panel B, Nat represents national level and Subnat represents subnational, Site represents site level

Figure 1 provides descriptive information on cost shares by country. Panel A presents the total national cost shares of routine infant immunization by administrative level. Site-level costs (including vaccine costs) represented 77–93% of total national costs. Panel B shows national, subnational and site level cost shares of service delivery (without vaccine costs) by budget category, and reveals variation between countries.

**Figure 2.**
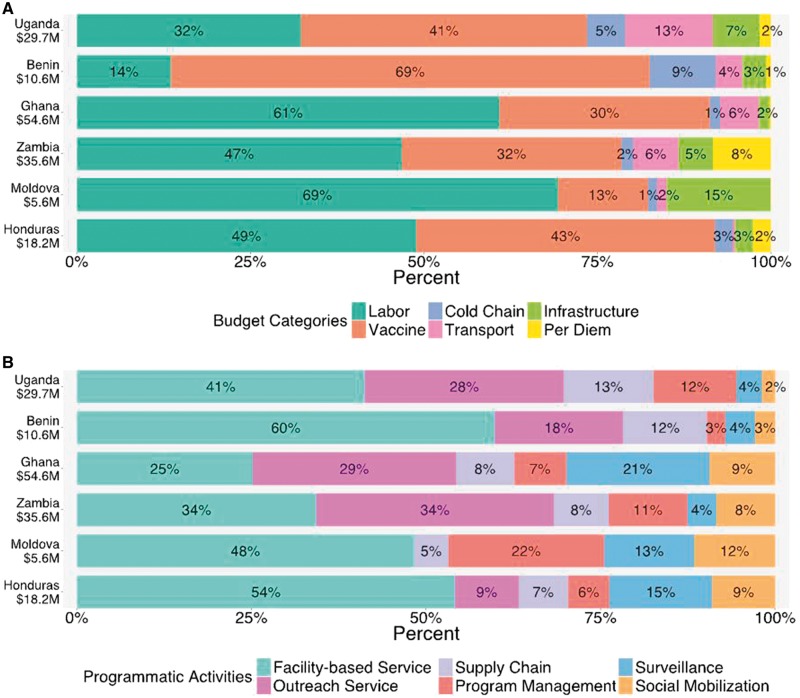
Share of total costs for infant routine immunization by budget category (Panel A) and programmatic activity (Panel B). Countries are displayed in increasing order of per capita GDP. Text shows percentage of costs in each category. Values for categories with percentage <0.5% are not shown. The numbers under countries’ names represent the estimated total facility level cost in each country, including vaccine costs

Figure 2 presents descriptive analyses for cost shares of site-level infant immunization in each country by budget category and programmatic activity. There was wide variation at this level between countries. By budget category, labour and vaccine costs formed the largest share of total costs, comprising 73–92% of the total cost across the six countries (mean = 83%). Of these two categories labour represented a larger share on average (mean = 45% for labour, 38% for vaccines). However, the relative share of costs for vaccines and labour varied widely between countries: in Moldova costs for labour were five times greater than for vaccines, while in Benin costs for vaccines were five times greater than for labour. By programmatic activity, the largest cost share was devoted to service delivery, either from fixed sites or via outreach, together comprising 48–78% of the total costs across six countries (mean = 63%). Facility-based costs were greater than outreach services costs in 4/6 countries. Social mobilization generally formed a greater percent of costs in countries with higher per capita GDP.

### Relationship between site characteristics and cost shares at site level

**Figure 3.**
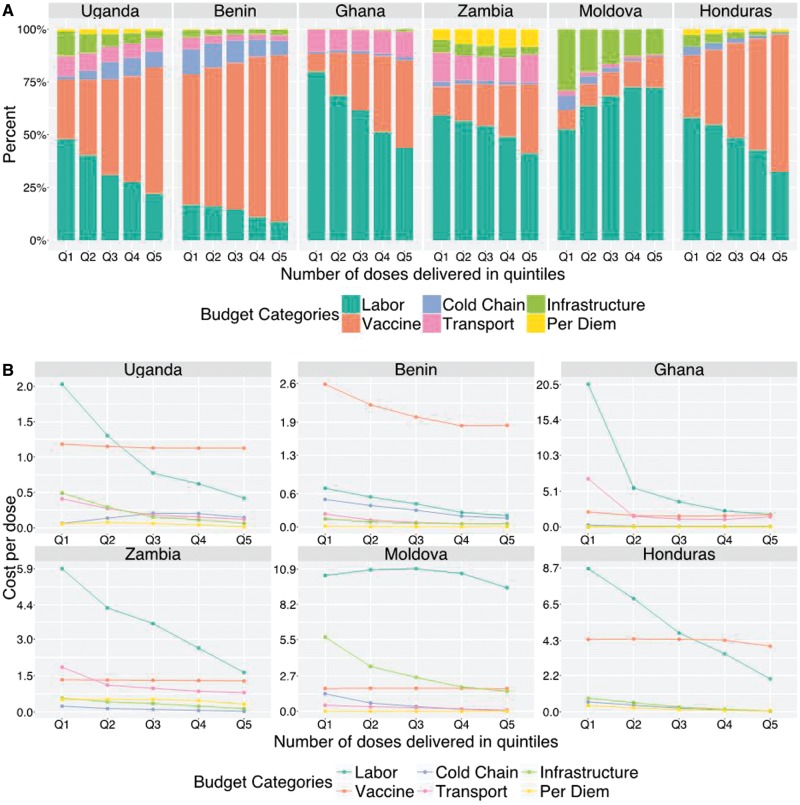
Changes in site-level cost shares (Panel A) and cost per dose (Panel B), across budget categories related to increasing service delivery volume. Bars within each country are displayed as increasing number of doses delivered, discretized by quintile. Percentages represent the weighted mean cost share of all facilities in each country

Regression analyses were used to investigate determinants of the cost share by budget category and programmatic activity within each country (coefficient estimates shown in Supplementary Table S3–S8 and S9–S14). Figure 3 (Panel A) shows how the cost shares at site level across budget categories change as a function of service volume (number of doses delivered), controlling for all the other effects in the regression equations (Supplementary Figure S1 shows these same results with vaccine costs removed). Panel B presents estimates for the cost per dose at site level for each budget category as a function of doses delivered. In general (with the exception of Moldova), increasing service volume is associated with a progressive increase in the cost share for vaccines and a reduction in the share of costs for other budget categories. From Panel B it is apparent that this trend results from reductions in non-vaccine costs per dose as service volume increases. The cost per dose for vaccines holds relatively stable as a function of service volume, with small variations in individual countries potentially indicating differences in wastage rates.

In Moldova, while there is a modest increase in the cost share for vaccines for sites with higher service volume, the major change related to service volume is a substantially greater share of costs for labour, which is offset by a reduction in the share of costs for buildings and other infrastructure. This appears due to reductions in buildings and cold chain costs as service volume increase, while other categories hold relatively flat.

**Figure 4.**
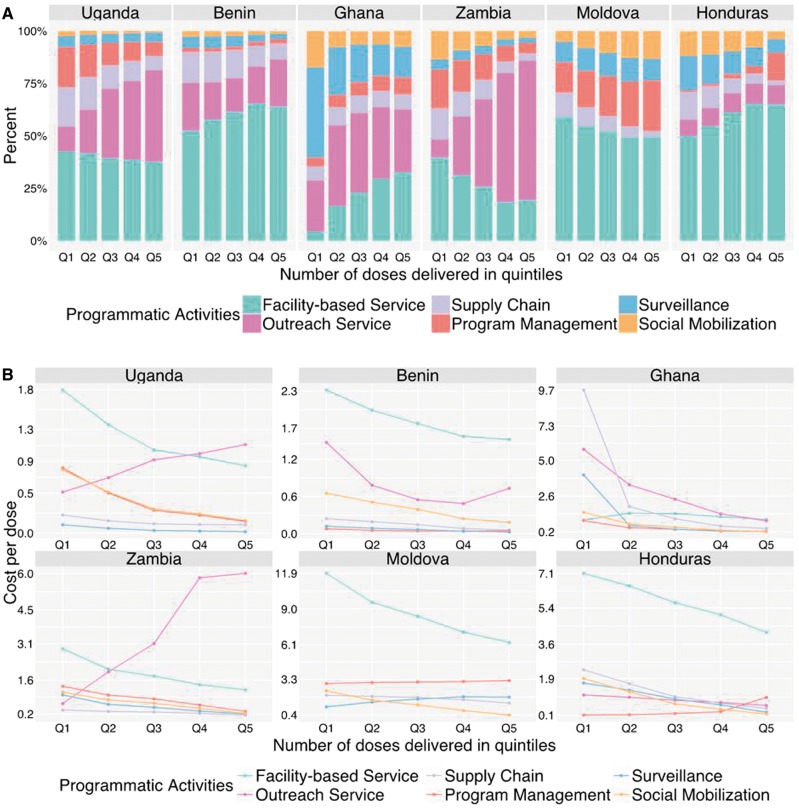
Changes in site-level cost shares (Panel A) and cost per dose (Panel B), across programmatic activity categories related to increasing services delivery volume. Bars within each country are displayed as increasing number of doses delivered, discretized by quintile. Percentages represent the weighted mean cost share of all facilities in each country

Figure 4 (Panel A) shows how costs at site level are distributed across programmatic activities as a function of service volume. In all countries expect Moldova, sites with a higher service volume experienced a higher share of costs devoted to service provision (facility-based or outreach). In Uganda and Zambia, this trend was associated with a substantially greater proportion of costs for outreach services. Figure 4 (Panel B) presents regression estimates for the cost per dose at site level for each programmatic activity as a function of doses delivered. In almost all countries and programmatic activity categories, the cost per dose decreases as service volume increases. Two major exceptions are in Uganda and Zambia, where costs for outreach services per dose is substantially higher for sites with higher service volume.

Supplementary Tables S15 and S16 present results for differences in the cost shares related to other cost determinants (area type, facility scale, ownership and distance to vaccine collection point). Although some of these comparisons are statistically significant there are few systematic trends across countries. In four out of six countries non-rural sites had a greater cost share devoted to labour compared with rural sites, although this difference was only statistically significant in Benin and Honduras. By programmatic activity, non-rural sites in Benin, Ghana, Moldova and Honduras devoted a higher cost share to surveillance, compared with rural sites (statistically significant in all four countries). 

### Relationship between site efficiency and cost shares

**Figure 5.**
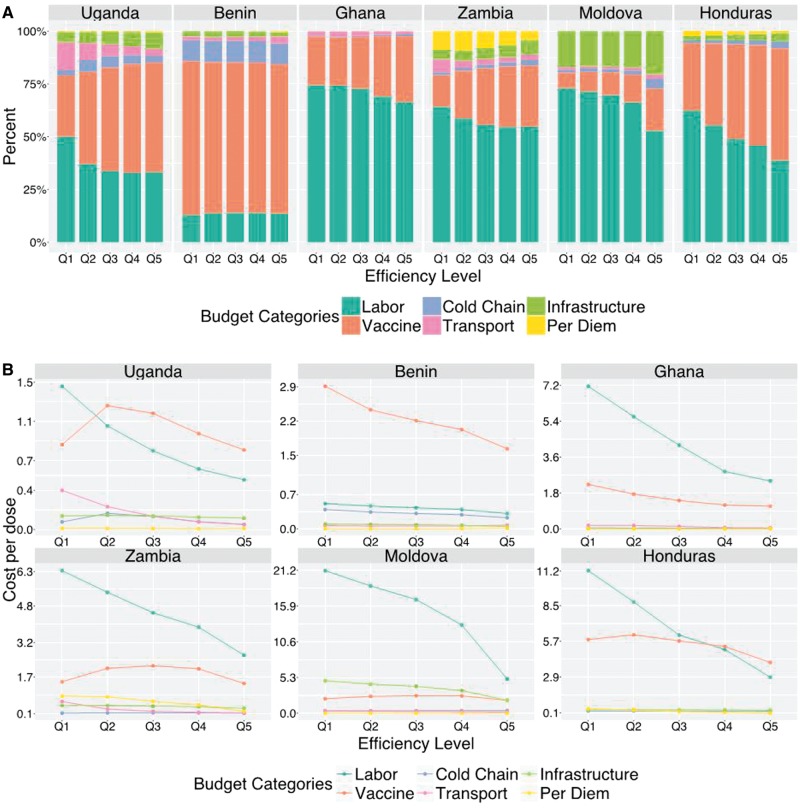
Changes in the site-level cost shares (Panel A) and cost per dose (Panel B), across budget categories related to increasing efficiency index. Bars within each country are displayed as increasing number of doses delivered, discretized by quintile. Percentages represent the weighted mean cost share of all facilities in each country

Figure 5 (Panel A) shows the cost shares across budget categories for different levels of the efficiency index. Within the same country, sites with a lower efficiency index (e.g. Q1 in Figure 5) have higher costs, controlling for service volume, area type, facility scale, ownership, and distance to vaccine collection point. With the exception of Benin, in each country the more efficient sites (according to the index) devoted a lower cost share to labour and a higher share to vaccines. Figure 5 (Panel B) presents the estimated cost per dose for each budget category as a function of increasing efficiency index. In general, the costs per dose are declining for increasing values of the efficiency index across all budget categories.

Across all six countries, more efficient sites devoted a greater cost share to providing facility-based services, and the cost per dose declines as a function of the efficiency index across almost all countries and programmatic activity categories (Supplementary Figure S2). Coefficient estimates of models are shown in Supplementary Tables S17–S29)

## Discussion

Using a unique dataset on immunization costs from six countries, these analyses provide a detailed description of the cost structure of routine infant immunizations services provided in different countries. The results of these analyses reveal substantial variation in the cost shares of immunization service between countries, and identify systematic trends in cost shares as a function of service delivery volume and site efficiency. Such information provides a better understanding of the resources required to provide immunization services.

In our analyses we allocated vaccine costs to the site-level. These analyses showed the fraction of national routine infant immunization costs that were incurred at the site-level to vary from 77 to 93% across countries. Even if vaccine costs are excluded, resources expended at site-level accounted for 29–81% of total national costs. At site-level, labour and vaccines comprised the largest proportion of costs (73–92%), yet the relative share of these two categories varied greatly. For example, in Moldova the ratio of labour to vaccine costs was 26 times higher than in Benin. Similar variability was observed in the smaller cost categories, with the cost share devoted to infrastructure in Moldova (15%) being twice as high as in any of the other six countries, and the cost share devoted to transport in Uganda (13%) being twice as high as in any of the other six countries. With vaccine costs excluded (Figure 1, Panel B), the fraction of costs devoted to cold chain was noticeably higher in Benin compared with other countries. Part of the reason for this could be lower costs incurred under other cost categories (particularly labour, for which Benin will have lower average salary levels compared with higher income countries in the sample). Another potential cause of this finding is inefficiency in the supply chain (Brown *et al.* 2014). Were these results the product of different unconnected studies, this variability might be attributable to differences in study methods. However, as the EPIC studies used consistent methods and tools in each country, this variability likely represents real differences between countries, related to variation in input prices, health system characteristics, and how the immunization programme is organized.

Given the substantial differences in cost shares between countries, regression analyses were conducted on a county-by-country basis to investigate how the cost shares correlated with observable site characteristics. By simultaneously controlling for multiple potential predictors, these analyses allowed us to estimate the individual effect of each predictor on site-level cost shares. From these analyses, sites with higher service volume (i.e. a higher reported number of doses delivered) had a lower share of costs attributable to labour and a higher share for vaccines, related to a rapid reduction in the labour cost per dose with increasing service volume (potentially due to more efficient use of labour at higher service volume). Although in most countries the vaccine cost per dose was relatively constant with increasing service volume, in Benin there was a 29% drop in the vaccine cost from the smallest quintile of doses delivered (median 2478 doses) to the largest quintile (median 13 276 doses). This decrease may be due to lower wastage for sites with higher service volume. Although the EPIC studies attempted to triangulate data on service volume, this finding is also consistent with measurement error in the reported number of doses delivered for each site. In each country the share of costs devoted to direct service provision increased with service volume. In Uganda and Zambia this was associated with a large increase in the cost share devoted to outreach, which was not evident in other countries. For almost all countries and cost categories, the cost share estimated for the lowest quintile was statistically significantly different to the cost share estimates for the highest quintile (Supplementary Tables S30 and S31). Consistent with operating in less-densely populated areas, rural sites devoted a greater cost share for outreach services, compared with non-rural sites.

We used an efficiency index to understand how differences in total costs were related to differences in the cost shares, for sites of similar service volume and other characteristics. These analyses showed that comparatively expensive sites (i.e. with a higher value of the efficiency index) had a higher cost per dose across most budget categories and programmatic activities. These differences were larger for labour costs, such that labour generally represented a greater share of costs in the more expensive sites. Similarly, the more expensive sites generally devoted a smaller share of resources to direct service provision. Statistical testing results for differences in the cost shares estimated for the sites with lowest and highest efficiency level by budget category and programatic activity were in Supplementary Tables 32 and 33. These differences in ‘efficiency’ need to be interpreted carefully. The variables controlled for when creating the efficiency index do not include all of the contextual factors that could influence the costs of providing immunization services, and so the differences described by this index will likely represent a mixture of factors that are amenable to intervention (such as time management by site personnel), and those that are not (such as ease of travel for the sites catchment population). In particular, these regressions did not include any measure of service quality, and therefore we cannot draw conclusions about the quality or health impact of immunization service delivery. Immunization coverage was another potentially important predictor we were not able to incorporate into the analysis, as estimates of target population size were found to include substantial measurement error. Unless introduced carefully, efforts to increase efficiency that do not take account of these broader concerns could induce perverse incentives or otherwise harm programmatic outcomes.

Additional limitations to this study include the potential misallocation of labour costs across programmatic activities, as reliance on reported (rather than observed) time allocation in the original surveys could potentially bias results if respondents were motivated to report a certain distribution of effort. Also, as we estimated the regression equations separately for each country the sample sizes for each equation were relatively small, limiting the ability of the analysis to identify small effects. Finally, the heterogeneity in cost shares between the six countries in the sample suggests caution in attempting to generalize these results to other countries.

The composition of immunization funding is likely to change as programmes add new vaccines and attempt to increase coverage, and as countries graduate from GAVI support. A detailed understanding of the cost structure of site-level service delivery costs helps programmes anticipate how funding changes might impact individual sites, what funding gaps might need to be filled is support is withdrawn from particular budget categories, what resources might be required to achieve higher service delivery volume, and potential areas where efficiencies could be pursued.

## Supplementary data

Supplementary data are available at *Health Policy and Planning* online.

## Supplementary Material

Supplementary Tables
