# Supplementary material for: The cost structure of routine infant immunization services: a systematic analysis of six countries
Source: Health Policy Plan. 2017 May 31;32(8):1174–84. doi: 10.1093/heapol/czx067 (PMC5886070; doi:10.1093/heapol/czx067)
Supplement: Supplementary Tables [file epic_cost_structure-supplement_revision_2.19.2017_czx067.docx]

Supplement for "The cost structure of routine infant immunization services: A systematic analysis of six countries". Geng et al, Health Policy and Planning

Table S1. EPIC study country Characteristics (Year 2011).

| Country | GDP per capita ^(1)^ | Infant Population | DTP3 Coverage Level ^(2)^ |
| --- | --- | --- | --- |
| Uganda | $598 | 1,326,826 | 80% |
| Benin | $799 | 348,577 | 85% |
| Ghana | $1,587 | 1,011,012 | 91% |
| Zambia | $1,636 | 567,320 | 81% |
| Moldova | $1,971 | 47,537 | 93% |
| Honduras | $2,308 | 177,733 | 91% |

1. World Bank national accounts data, and OECD National Accounts data files. Available at <http://databank.worldbank.org/data/reports.aspx?source=2&series=NY.GDP.PCAP.CD&country>=, accessed on Feb.3, 2017
2. WHO-UNICEF estimates of DTP3 coverage. DTP3 = Diptheria, Tetanus, Pertussis Vaccine, 3rd dose. Available at <http://apps.who.int/immunization_monitoring/globalsummary/timeseries/tswucoveragedtp3.html>, accessed on Apr.4, 2016

Table S2. Routine immunization Schedule for infant under 1 year old.

| Uganda | Benin | Ghana | Zambia | Moldova | Honduras |
| --- | --- | --- | --- | --- | --- |
| Birth | Birth | Birth | Birth | Birth | Birth |
| BCG, OPV0 | BCG, OPV0 | BCG, OPV0 | BCG, OPV0 | HepB | BCG, HepB |
| 6 weeks | 6 weeks | 6 weeks | 6 weeks | 2-5 days | 2 months |
| OPV1, DTP-HepB-Hib1 | OPV1, DTP-HepB-Hib1 | OPV1, Penta | OPV1, HepB-Hib1-DTP | BCG | OPV1, DTP-HepB-Hib1, Rota1 |
| 10 weeks | 10 weeks | 10 weeks | 10 weeks | 2 months | 4 months |
| DTP-HepB-Hib2, OPV2 | OPV2, DTP-HepB-Hib2 | OPV2,  Penta | OPV2, HepB-Hib2-DTP | DTP-Hib1, OPV1 | OPV2, DTP-HepB-Hib2, Rota2 |
| 14 weeks | 14 weeks | 14 weeks | 14 weeks | 4 months | 6 months |
| DTP-HepB-Hib2, OPV3 | OPV3, DTP-HepB-Hib2 | OPV3, Penta | OPV3, HepB-Hib2-DTP | DTP-Hib2, OPV2 | OPV3, DTP-HepB-Hib3 |
| 9 months | 9 months | 9 months | 9 months | 6 months | 12-23 months |
| Measles 1 | Measles 1, yellow fever | Measles 1, yellow fever | Measles | DTP-Hib3, OPV3 | MMR1,  YF (high risk) |

Table S3. Coefficient estimates and residual correlation for regression analysis of relationship between site characteristics and cost shares for each budget category (Uganda).

| Coefficient estimates for SUR regression equations (coefficient, (standard error)) | | | | | | |
| --- | --- | --- | --- | --- | --- | --- |
|  | Labor | Vaccine | Cold Chain | Vehicle | Infrastructure | Per Diem |
| (Intercept) | 13.14 *** | 8.74 *** | -24.54 * | 11.63 | 11.05 *** | -41.73 * |
|  | (<0.001) | (<0.001) | (<0.01) | (0.06) | (<0.001) | (0.03) |
| Rural | -0.13 | 0.06 | 0.08 | 0.24 | 0.17 | 0.91 |
|  | (0.50) | (0.09) | (0.91) | (0.63) | (0.47) | (0.56) |
| Hospital | 0.85 | 0.02 | -0.16 | 1.68 | 0.22 | 2.77 |
|  | (<0.001) | (0.63) | (0.83) | (<0.01) | (0.39) | (0.11) |
| Government-owned | 0.85 | 0.01 | -0.03 | -1.63 * | -0.73 * | -0.07 |
|  | (0.83) | (0.81) | (0.96) | (<0.01) | (<0.01) | (0.96) |
| Distance | 0.00 | 0.00 | 0.02 | 0.04 * | 0.00 | -0.01 |
|  | (0.93) | (0.42) | (0.48) | (0.02) | (0.91) | (0.82) |
| log(Number of doses) | 0.22 | 0.81 *** | 8.30 *** | 0.05 | 0.61 | 12.48 * |
|  | (0.70) | (<0.001) | (<0.001) | (0.97) | (0.38) | (<0.01) |
| log(Number of doses)^2^ | 0.01 | 0.01 | -0.44 *** | 0.03 | -0.02 | -0.74 * |
|  | (0.75) | (0.09) | (<0.001) | (0.74) | (0.58) | (0.01) |
| Adjusted R-squared | 0.66 | 0.99 | 0.45 | 0.45 | 0.23 | 0.14 |
| Correlation matrix for SUR regression residuals | | | | | | |
|  | Labor | Vaccine | Cold Chain | Vehicle | Infrastructure | Per Diem |
| Labor | 1.00 | 0.15 | -0.07 | 0.27 | -0.20 | -0.14 |
| Vaccine | 0.15 | 1.00 | -0.17 | -0.11 | -0.03 | -0.09 |
| Cold Chain | -0.07 | -0.17 | 1.00 | 0.01 | 0.18 | 0.16 |
| Vehicle | 0.27 | -0.11 | 0.01 | 1.00 | 0.02 | 0.08 |
| Infrastructure | -0.20 | -0.03 | 0.18 | 0.02 | 1.00 | 0.10 |
| Per Diem | -0.14 | -0.09 | 0.16 | 0.08 | 0.10 | 1.00 |
| McElroy R-squared (goodness of fit for system of equations) = 0.97. | | | | | | |
| *** Indicates statistical significance with p<0.001. * Indicates statistical significance with p<0.05. | | | | | | |

Table S4. Coefficient estimates and residual correlation for regression analysis of relationship between site characteristics and cost shares for each budget category (Benin).

| Coefficient estimates for SUR regression equations (coefficient, (standard error)) | | | | | | |
| --- | --- | --- | --- | --- | --- | --- |
|  | Labor | Vaccine | Cold Chain | Vehicle | Infrastructure | Per Diem |
| (Intercept) | 6.77 | 20.10 * | 8.41 | 53.40 * | 42.64 | 85.52 |
|  | (0.60) | (<0.01) | (0.57) | (0.05) | (0.06) | (0.27) |
| Rural | -0.92 *** | -0.10 | -0.14 | 0.44 | 0.61 | 1.20 |
|  | (<0.001) | (0.32) | (0.54) | (0.28) | (0.08) | (0.31) |
| Government-owned | 1.15 *** | 0.02 | -0.18 | 1.28 | -0.92 | 5.02 * |
|  | (<0.001) | (0.91) | (0.61) | (0.05) | (0.10) | (0.01) |
| Distance | -0.01 | 0.00 | 0.00 | 0.03 * | 0.01 | -0.04 |
|  | (0.18) | (0.96) | (0.64) | (<0.01) | (0.09) | (0.15) |
| log(Number of doses) | 1.28 | -1.85 | 0.95 | -10.28 | -7.49 | -19.11 |
|  | (0.67) | (0.22) | (0.78) | (0.10) | (0.15) | (0.29) |
| log(Number of doses)^2^ | -0.06 | 0.15 | -0.04 | 0.60 | 0.46 | 1.11 |
|  | (0.74) | (0.09) | (0.85) | (0.10) | (0.14) | (0.30) |
| Adjusted R-squared | 0.56 | 0.81 | 0.05 | 0.32 | 0.10 | 0.19 |
| Correlation matrix for SUR regression residuals | | | | | | |
|  | Labor | Vaccine | Cold Chain | Vehicle | Infrastructure | Per Diem |
| Labor | 1.00 | -0.12 | 0.23 | -0.26 | 0.01 | -0.06 |
| Vaccine | -0.12 | 1.00 | 0.09 | 0.15 | 0.07 | -0.21 |
| Cold Chain | 0.23 | 0.09 | 1.00 | -0.25 | -0.08 | -0.24 |
| Vehicle | -0.26 | 0.15 | -0.25 | 1.00 | 0.29 | 0.52 |
| Infrastructure | 0.01 | 0.07 | -0.08 | 0.29 | 1.00 | 0.45 |
| Per Diem | -0.06 | -0.21 | -0.24 | 0.52 | 0.45 | 1.00 |
| McElroy R-squared (goodness of fit for system of equations) = 0.62. | | | | | | |
| *** Indicates statistical significance with p<0.001. * Indicates statistical significance with p<0.05. | | | | | | |

Table S5. Coefficient estimates and residual correlation for regression analysis of relationship between site characteristics and cost shares for each budget category (Ghana).

| Coefficient estimates for SUR regression equations (coefficient, (standard error)) | | | | | | |
| --- | --- | --- | --- | --- | --- | --- |
|  | Labor | Vaccine | Cold Chain | Vehicle | Infrastructure | Per Diem |
| (Intercept) | 18.10 *** | 7.58 | 4.99 | 18.69 | 4.77 | 5.19 |
|  | (<0.001) | (0.09) | (0.74) | (0.35) | (0.78) | (0.67) |
| Rural | -0.48 * | -0.08 | 0.26 | 0.79 | 1.22 | -0.47 |
|  | (0.02) | (0.73) | (0.73) | (0.42) | (0.15) | (0.43) |
| Hospital | -0.08 | -0.49 | -1.26 | -3.54 | -2.70 | -0.99 |
|  | (0.83) | (0.24) | (0.37) | (0.06) | (0.10) | (0.39) |
| Government-owned | 0.05 | -0.58 | -1.50 | -4.32 | -2.87 | -0.41 |
|  | (0.92) | (0.29) | (0.41) | (0.08) | (0.17) | (0.78) |
| Distance | 0.02 * | 0.01 | 0.04 | 0.02 | 0.02 | -0.01 |
|  | (0.01) | (0.14) | (0.20) | (0.61) | (0.55) | (0.77) |
| log(Number of doses) | -2.37 * | -0.57 | -0.26 | -2.95 | -1.54 | -0.92 |
|  | (0.02) | (0.62) | (0.95) | (0.57) | (0.73) | (0.77) |
| log(Number of doses)^2^ | 0.16 * | 0.10 | 0.05 | 0.22 | 0.20 | 0.09 |
|  | (0.02) | (0.21) | (0.85) | (0.52) | (0.50) | (0.67) |
| Adjusted R-squared | 0.26 | 0.64 | 0.00 | 0.04 | 0.17 | -0.05 |
| Correlation matrix for SUR regression residuals | | | | | | |
|  | Labor | Vaccine | Cold Chain | Vehicle | Infrastructure | Per Diem |
| Labor | 1.00 | 0.13 | 0.09 | 0.02 | 0.21 | 0.09 |
| Vaccine | 0.13 | 1.00 | 0.04 | -0.20 | 0.08 | -0.12 |
| Cold Chain | 0.09 | 0.04 | 1.00 | -0.22 | 0.42 | 0.11 |
| Vehicle | 0.02 | -0.20 | -0.22 | 1.00 | 0.08 | 0.19 |
| Infrastructure | 0.21 | 0.08 | 0.42 | 0.08 | 1.00 | 0.02 |
| Per Diem | 0.09 | -0.12 | 0.11 | 0.19 | 0.02 | 1.00 |
| McElroy R-squared (goodness of fit for system of equations) = 0.37. | | | | | | |
| *** Indicates statistical significance with p<0.001. * Indicates statistical significance with p<0.05. | | | | | | |

Table S6. Coefficient estimates and residual correlation for regression analysis of relationship between site characteristics and cost shares for each budget category (Zambia).

| Coefficient estimates for SUR regression equations (coefficient, (standard error)) | | | | | | |
| --- | --- | --- | --- | --- | --- | --- |
|  | Labor | Vaccine | Cold Chain | Vehicle | Infrastructure | Per Diem |
| (Intercept) | 13.71 *** | 8.70 *** | 15.74 * | 16.58 | 11.25 * | -1.65 |
|  | (<0.001) | (<0.001) | (<0.01) | (0.53) | (<0.01) | (0.87) |
| Rural | 0.69 *** | 0.02 | 0.08 | 3.70 * | 0.38 * | 0.90 |
|  | (<0.001) | (0.49) | (0.77) | (<0.01) | (0.05) | (0.07) |
| Hospital | 0.35 | -0.01 | 0.53 | -3.06 | 0.49 * | 1.00 |
|  | (0.15) | (0.89) | (0.12) | (0.07) | (0.05) | (0.12) |
| Government-owned | 0.13 | 0.11 | -0.35 | -1.45 | -0.29 | -1.16 |
|  | (0.70) | (0.06) | (0.46) | (0.54) | (0.40) | (0.20) |
| Distance | 0.00 | 0.00 | 0.00 | 0.01 | 0.00 | 0.00 |
|  | (0.51) | (0.69) | (0.40) | (0.68) | (0.43) | (0.48) |
| log(Number of doses) | 0.42 | 1.00 *** | -0.49 | -1.25 | 0.65 | 3.56 |
|  | (0.63) | (<0.001) | (0.69) | (0.83) | (0.47) | (0.12) |
| log(Number of doses)^2^ | 0.00 | 0.00 | 0.04 | 0.11 | -0.01 | -0.16 |
|  | (0.97) | (0.90) | (0.55) | (0.76) | (0.79) | (0.21) |
| Adjusted R-squared | 0.43 | 0.99 | 0.07 | 0.21 | 0.39 | 0.23 |
| Correlation matrix for SUR regression residuals | | | | | | |
|  | Labor | Vaccine | Cold Chain | Vehicle | Infrastructure | Per Diem |
| Labor | 1.00 | 0.14 | -0.08 | 0.09 | 0.06 | 0.23 |
| Vaccine | 0.14 | 1.00 | 0.01 | -0.11 | 0.26 | 0.14 |
| Cold Chain | -0.08 | 0.01 | 1.00 | -0.21 | 0.21 | 0.10 |
| Vehicle | 0.09 | -0.11 | -0.21 | 1.00 | -0.21 | 0.17 |
| Infrastructure | 0.06 | 0.26 | 0.21 | -0.21 | 1.00 | 0.22 |
| Per Diem | 0.23 | 0.14 | 0.10 | 0.17 | 0.22 | 1.00 |
| McElroy R-squared (goodness of fit for system of equations) = 0.97. | | | | | | |
| *** Indicates statistical significance with p<0.001. * Indicates statistical significance with p<0.05. | | | | | | |

Table S7. Coefficient estimates and residual correlation for regression analysis of relationship between site characteristics and cost shares for each budget category (Moldova).

| Coefficient estimates for SUR regression equations (coefficient, (standard error)) | | | | | | |
| --- | --- | --- | --- | --- | --- | --- |
|  | Labor | Vaccine | Cold Chain | Vehicle | Infrastructure | Per Diem |
| (Intercept) | 4.22 *** | 2.98 *** | 4.99 | 18.69 | 4.77 | 5.19 |
|  | (<0.001) | (<0.001) | (0.74) | (0.35) | (0.78) | (0.67) |
| Rural | -0.17 | -0.03 | 0.26 | 0.79 | 1.22 | -0.47 |
|  | (0.70) | (0.77) | (0.73) | (0.42) | (0.15) | (0.43) |
| Distance | 0.01 | 0.00 | 0.04 | 0.02 | 0.02 | -0.01 |
|  | (0.39) | (0.44) | (0.20) | (0.61) | (0.55) | (0.77) |
| log(Number of doses) | 1.27 * | 1.04 *** | -0.26 | -2.95 | -1.54 | -0.92 |
|  | (<0.01) | (<0.001) | (0.95) | (0.57) | (0.73) | (0.77) |
| log(Number of doses)^2^ | -0.03 | 0.00 | 0.05 | 0.22 | 0.20 | 0.09 |
|  | (0.53) | (0.68) | (0.85) | (0.52) | (0.50) | (0.67) |
| Adjusted R-squared | 0.84 | 0.99 | 0.00 | 0.04 | 0.17 | -0.05 |
| Correlation matrix for SUR regression residuals | | | | | | |
|  | Labor | Vaccine | Cold Chain | Vehicle | Infrastructure | Per Diem |
| Labor | 1.00 | 0.26 | -0.11 | 0.17 | 0.56 | -0.06 |
| Vaccine | 0.26 | 1.00 | 0.14 | 0.07 | 0.37 | -0.23 |
| Cold Chain | -0.11 | 0.14 | 1.00 | -0.11 | -0.11 | -0.02 |
| Vehicle | 0.17 | 0.07 | -0.11 | 1.00 | 0.19 | 0.28 |
| Infrastructure | 0.56 | 0.37 | -0.11 | 0.19 | 1.00 | 0.06 |
| Per Diem | -0.06 | -0.23 | -0.02 | 0.28 | 0.06 | 1.00 |
| McElroy R-squared (goodness of fit for system of equations) = 0.96. | | | | | | |
| *** Indicates statistical significance with p<0.001. * Indicates statistical significance with p<0.05. | | | | | | |

Table S8. Coefficient estimates and residual correlation for regression analysis of relationship between site characteristics and cost shares for each budget category (Honduras).

| Coefficient estimates for SUR regression equations (coefficient, (standard error)) | | | | | | | |
| --- | --- | --- | --- | --- | --- | --- | --- |
|  | Labor | Vaccine | Cold Chain | | Infrastructure | Per Diem | |
| (Intercept) | 9.44 *** | 3.97 *** | 7.67 *** | | 8.65 *** | 11.98 * | |
|  | (<0.001) | (<0.001) | (<0.001) | | (<0.001) | (0.02) | |
| Rural | -0.43 * | -0.03 | 0.19 | | 0.00 | 0.09 | |
|  | (<0.01) | (0.31) | (0.31) | | (0.98) | (0.86) | |
| Hospital | -0.13 | -0.57 *** | 0.02 | | -0.13 | 0.03 | |
|  | (0.73) | (<0.001) | (0.96) | | (0.74) | (0.98) | |
| log(Number of doses) | 0.15 | 1.15 *** | -0.04 | | -0.15 | -1.46 | |
|  | (0.73) | (<0.001) | (0.94) | | (0.73) | (0.30) | |
| log(Number of doses)^2^ | 0.03 | -0.01 | 0.02 | | 0.03 | 0.13 | |
|  | (0.33) | (0.07) | (0.57) | | (0.39) | (0.18) | |
| Adjusted R-squared | 0.73 | 0.99 | 0.21 | | 0.24 | 0.09 | |
| Correlation matrix for SUR regression residuals | | | | | | | |
|  | Labor | Vaccine | Cold Chain | | Infrastructure | Per Diem | |
| Labor | 1.00 | 0.23 | -0.16 | | -0.15 | 0.35 | |
| Vaccine | 0.23 | 1.00 | 0.08 | | 0.21 | 0.13 | |
| Cold Chain | -0.16 | 0.08 | 1.00 | | 0.22 | 0.03 | |
| Infrastructure | -0.15 | 0.21 | 0.22 | | 1.00 | 0.03 | |
| Per Diem | 0.35 | 0.13 | 0.03 | | 0.03 | 1.00 | |
| McElroy R-squared (goodness of fit for system of equations) = 0.97. | | | |  | | |  |
| *** Indicates statistical significance with p<0.001. * Indicates statistical significance with p<0.05. | | | | | | | |

Table S9. Coefficient estimates and residual correlation for regression analysis of relationship between site characteristics and cost shares for each programmatic activity (Uganda).

| Coefficient estimates for SUR regression equations (coefficient, (standard error)) | | | | | | |
| --- | --- | --- | --- | --- | --- | --- |
|  | Facility-based service | Surveillance | Program Management | Outreach service | Social Mobilization | Supply Chain |
| (Intercept) | 13.71 *** | 12.18 | 12.50 *** | -2.13 | 9.11 | 11.41 *** |
|  | (<0.001) | (0.22) | (<0.001) | (0.82) | (0.33) | (<0.001) |
| Rural | -0.07 | 0.79 | 0.20 | -0.20 | -0.81 | -0.05 |
|  | (0.41) | (0.34) | (0.38) | (0.80) | (0.30) | (0.79) |
| Hospital | 0.35 * | -0.34 | 0.99 *** | 0.16 | 0.56 | 0.77 *** |
|  | (<0.01) | (0.70) | (<0.001) | (0.85) | (0.50) | (<0.001) |
| Government-owned | -0.24 * | -0.23 | -0.43 | -0.34 | 0.87 | -0.39 |
|  | (0.02) | (0.79) | (0.08) | (0.67) | (0.28) | (0.06) |
| Distance | 0.00 | 0.01 | 0.00 | 0.02 | 0.02 | 0.01 |
|  | (0.20) | (0.82) | (0.60) | (0.53) | (0.42) | (0.08) |
| log(Number of doses) | -0.13 | -0.46 | 0.20 | 3.14 | 0.33 | 0.47 |
|  | (0.63) | (0.85) | (0.77) | (0.16) | (0.88) | (0.43) |
| log(Number of doses)^2^ | 0.05 * | 0.07 | 0.01 | -0.12 | 0.00 | -0.01 |
|  | (<0.01) | (0.64) | (0.83) | (0.40) | (0.99) | (0.89) |
| Adjusted R-squared | 0.94 | 0.99 | 0.51 | 0.33 | 0.23 | 0.63 |
| Correlation matrix for SUR regression residuals | | | | | | |
|  | Facility-based service | Surveillance | Program Management | Outreach service | Social Mobilization | Supply Chain |
| Facility-based service | 1.00 | -0.08 | 0.21 | -0.09 | 0.03 | 0.36 |
| Surveillance | -0.08 | 1.00 | 0.40 | 0.09 | 0.19 | 0.24 |
| Program Management | 0.21 | 0.40 | 1.00 | 0.27 | 0.33 | 0.51 |
| Outreach service | -0.09 | 0.09 | 0.27 | 1.00 | 0.14 | 0.01 |
| Social Mobilization | 0.03 | 0.19 | 0.33 | 0.14 | 1.00 | 0.22 |
| Supply Chain | 0.36 | 0.24 | 0.51 | 0.01 | 0.22 | 1.00 |
| McElroy R-squared (goodness of fit for system of equations) = 0.77. | | | | | | |
| *** Indicates statistical significance with p<0.001. * Indicates statistical significance with p<0.05. | | | | | | |

Table S10. Coefficient estimates and residual correlation for regression analysis of relationship between site characteristics and cost shares for each programmatic activity (Benin).

| Coefficient estimates for SUR regression equations (coefficient, (standard error)) | | | | | | |
| --- | --- | --- | --- | --- | --- | --- |
|  | Facility-based service | Surveillance | Program Management | Outreach service | Social Mobilization | Supply Chain |
| (Intercept) | 17.34 * | -4.45 | 43.05 | 49.35 | 7.18 | 4.76 |
|  | (0.02) | (0.76) | (0.12) | (0.46) | (0.74) | (0.71) |
| Rural | -0.23 * | -1.17 *** | -0.40 | 1.98 | -0.27 | -0.24 |
|  | (0.04) | (<0.001) | (0.34) | (0.06) | (0.43) | (0.23) |
| Government-owned | -0.18 | 1.52 *** | 1.05 | 4.39 * | 1.30 * | 0.02 |
|  | (0.32) | (<0.001) | (0.13) | (0.01) | (0.02) | (0.96) |
| Distance | 0.00 | -0.01 | 0.01 | 0.03 | 0.01 | <0.001 |
|  | (0.15) | (0.29) | (0.25) | (0.32) | (0.13) | (0.93) |
| log(Number of doses) | -1.11 | 3.68 | -8.20 | -10.34 | 0.61 | 1.88 |
|  | (0.52) | (0.29) | (0.21) | (0.51) | (0.91) | (0.53) |
| log(Number of doses)^2^ | 0.11 | -0.20 | 0.51 | 0.63 | -0.02 | -0.09 |
|  | (0.29) | (0.32) | (0.18) | (0.50) | (0.94) | (0.59) |
| Adjusted R-squared | 0.76 | 0.55 | 0.22 | 0.26 | 0.15 | 0.09 |
| Correlation matrix for SUR regression residuals | | | | | | |
|  | Facility-based service | Surveillance | Program Management | Outreach service | Social Mobilization | Supply Chain |
| Facility-based service | 1.00 | 0.25 | -0.14 | -0.07 | -0.11 | 0.01 |
| Surveillance | 0.25 | 1.00 | -0.11 | 0.03 | -0.08 | 0.30 |
| Program Management | -0.14 | -0.11 | 1.00 | 0.21 | 0.25 | 0.13 |
| Outreach service | -0.07 | 0.03 | 0.21 | 1.00 | 0.16 | 0.16 |
| Social Mobilization | -0.11 | -0.08 | 0.25 | 0.16 | 1.00 | -0.16 |
| Supply Chain | 0.01 | 0.30 | 0.13 | 0.16 | -0.16 | 1.00 |
| McElroy R-squared (goodness of fit for system of equations) = 0.52. | | | | | | |
| *** Indicates statistical significance with p<0.001. * Indicates statistical significance with p<0.05. | | | | | | |

Table S11. Coefficient estimates and residual correlation for regression analysis of relationship between site characteristics and cost shares for each programmatic activity (Ghana).

| Coefficient estimates for SUR regression equations (coefficient, (standard error)) | | | | | | |
| --- | --- | --- | --- | --- | --- | --- |
|  | Facility-based service | Surveillance | Program Management | Outreach service | Social Mobilization | Supply Chain |
| (Intercept) | 19.73 *** | 12.18 | 5.28 | -2.62 | 28.39 *** | 2.65 |
|  | (<0.001) | (0.22) | (0.52) | (0.83) | (<0.001) | (0.70) |
| Rural | -0.81 *** | 0.79 | -0.24 | 0.73 | -0.43 | 0.04 |
|  | (<0.01) | (0.34) | (0.55) | (0.23) | (0.27) | (0.91) |
| Hospital | 0.02 | -0.34 | 0.13 | -1.19 | -0.74 | -0.23 |
|  | (0.97) | (0.70) | (0.86) | (0.31) | (0.32) | (0.72) |
| Government-owned | 0.58 | -0.23 | 0.61 | -1.25 | -1.08 | -1.62 |
|  | (0.37) | (0.79) | (0.54) | (0.40) | (0.26) | (0.05) |
| Distance | 0.02 | 0.01 | 0.03 | 0.02 | 0.04 * | 0.01 |
|  | (0.15) | (0.82) | (0.12) | (0.51) | (0.02) | (0.33) |
| log(Number of doses) | -2.92 * | -0.46 | -0.21 | 2.86 | -5.37 * | 1.36 |
|  | (0.04) | (0.85) | (0.92) | (0.37) | (0.01) | (0.44) |
| log(Number of doses)^2^ | 0.18 | 0.07 | 0.05 | -0.17 | 0.35 * | -0.07 |
|  | (0.05) | (0.64) | (0.75) | (0.43) | (0.01) | (0.54) |
| Adjusted R-squared | 0.19 | 0.99 | 0.10 | 0.02 | 0.19 | 0.14 |
| Correlation matrix for SUR regression residuals | | | | | | |
|  | Facility-based service | Surveillance | Program Management | Outreach service | Social Mobilization | Supply Chain |
| Facility-based service | 1.00 | 0.11 | 0.34 | -0.14 | 0.20 | 0.31 |
| Surveillance | 0.11 | 1.00 | 0.48 | -0.14 | 0.18 | 0.02 |
| Program Management | 0.34 | 0.48 | 1.00 | -0.11 | 0.23 | 0.35 |
| Outreach service | -0.14 | -0.14 | -0.11 | 1.00 | -0.01 | 0.04 |
| Social Mobilization | 0.20 | 0.18 | 0.23 | -0.01 | 1.00 | 0.20 |
| Supply Chain | 0.31 | 0.02 | 0.35 | 0.04 | 0.20 | 1.00 |
| McElroy R-squared (goodness of fit for system of equations) = 0.24. | | | | | | |
| *** Indicates statistical significance with p<0.001. * Indicates statistical significance with p<0.05. | | | | | | |

Table S12. Coefficient estimates and residual correlation for regression analysis of relationship between site characteristics and cost shares for each programmatic activity (Zambia).

| Coefficient estimates for SUR regression equations (coefficient, (standard error)) | | | | | | |
| --- | --- | --- | --- | --- | --- | --- |
|  | Facility-based service | Surveillance | Program Management | Outreach service | Social Mobilization | Supply Chain |
| (Intercept) | 20.88 *** | 5.41 | 9.69 | -47.25 * | 18.22 | 9.19 |
|  | (<0.001) | (0.50) | (0.09) | (0.02) | (0.08) | (0.08) |
| Rural | 0.29 | 1.23 * | 0.67 * | 0.63 | 0.38 | 1.09 *** |
|  | (0.08) | (<0.01) | (0.02) | (0.52) | (0.45) | (<0.001) |
| Hospital | 0.23 | 0.25 | 0.34 | -0.34 | 0.65 | 0.55 |
|  | (0.27) | (0.62) | (0.34) | (0.79) | (0.33) | (0.10) |
| Government-owned | -0.35 | -0.15 | -0.50 | 0.31 | -0.21 | -0.06 |
|  | (0.24) | (0.84) | (0.32) | (0.86) | (0.82) | (0.89) |
| Distance | 0.00 | 0.00 | 0.00 | 0.01 | 0.00 | 0.00 |
|  | (0.54) | (0.47) | (0.20) | (0.52) | (0.96) | (0.68) |
| log(Number of doses) | -1.45 | 1.61 | 1.28 | 13.46 * | -0.94 | 1.15 |
|  | (0.06) | (0.38) | (0.32) | (<0.01) | (0.69) | (0.33) |
| log(Number of doses)^2^ | 0.12 * | -0.06 | -0.05 | -0.69 * | 0.08 | -0.05 |
|  | (<0.01) | (0.58) | (0.46) | (0.01) | (0.57) | (0.46) |
| Adjusted R-squared | 0.76 | 0.20 | 0.17 | 0.21 | -0.01 | 0.35 |
| Correlation matrix for SUR regression residuals | | | | | | |
|  | Facility-based service | Surveillance | Program Management | Outreach service | Social Mobilization | Supply Chain |
| Facility-based service | 1.00 | 0.39 | 0.25 | 0.06 | -0.20 | 0.01 |
| Surveillance | 0.39 | 1.00 | 0.56 | 0.07 | 0.06 | 0.43 |
| Program Management | 0.25 | 0.56 | 1.00 | -0.01 | -0.11 | 0.37 |
| Outreach service | 0.06 | 0.07 | -0.01 | 1.00 | 0.12 | 0.18 |
| Social Mobilization | -0.20 | 0.06 | -0.11 | 0.12 | 1.00 | -0.06 |
| Supply Chain | 0.01 | 0.43 | 0.37 | 0.18 | -0.06 | 1.00 |
| McElroy R-squared (goodness of fit for system of equations) = 0.5. | | | | | | |
| *** Indicates statistical significance with p<0.001. * Indicates statistical significance with p<0.05. | | | | | | |

Table S13. Coefficient estimates and residual correlation for regression analysis of relationship between site characteristics and cost shares for each programmatic activity (Moldova).

| Coefficient estimates for SUR regression equations (coefficient, (standard error)) | | | | | | | |
| --- | --- | --- | --- | --- | --- | --- | --- |
|  | Facility-based service | Surveillance | Program Management | Social Mobilization | | Supply Chain | |
| (Intercept) | 5.97 *** | 3.42 * | 3.30 * | 0.05 | | 4.33 *** | |
|  | (<0.001) | (<0.01) | (<0.01) | (0.98) | | (<0.001) | |
| Rural | 0.06 | -0.70 | -0.23 | 0.04 | | -0.14 | |
|  | (0.82) | (0.11) | (0.63) | (0.96) | | (0.73) | |
| Distance | 0.00 | 0.01 | 0.01 | -0.01 | | 0.01 | |
|  | (0.58) | (0.14) | (0.31) | (0.56) | | (0.08) | |
| log(Number of doses) | 0.58 * | 1.10 * | 1.11 * | 2.02 * | | 0.70 | |
|  | (0.03) | (0.02) | (0.03) | (0.02) | | (0.10) | |
| log(Number of doses)^2^ | 0.02 | -0.02 | -0.01 | -0.08 | | -0.01 | |
|  | (0.34) | (0.67) | (0.85) | (0.32) | | (0.73) | |
| Adjusted R-squared | 0.92 | 0.85 | 0.83 | 0.64 | | 0.63 | |
| Correlation matrix for SUR regression residuals | | | | | | | |
|  | Facility-based service | Surveillance | Program Management | Social Mobilization | | Supply Chain | |
| Facility-based service | 1.00 | 0.67 | 0.77 | 0.56 | | 0.45 | |
| Surveillance | 0.67 | 1.00 | 0.84 | 0.70 | | 0.61 | |
| Program Management | 0.77 | 0.84 | 1.00 | 0.61 | | 0.62 | |
| Social Mobilization | 0.56 | 0.70 | 0.61 | 1.00 | | 0.43 | |
| Supply Chain | 0.45 | 0.61 | 0.62 | 0.43 | | 1.00 | |
| McElroy R-squared (goodness of fit for system of equations) = 0.75. | | | | |  | |  |
| *** Indicates statistical significance with p<0.001. * Indicates statistical significance with p<0.05. | | | | | | | |

Table S14. Coefficient estimates and residual correlation for regression analysis of relationship between site characteristics and cost shares for each programmatic activity (Honduras).

| Coefficient estimates for SUR regression equations (coefficient, (standard error)) | | | | | | |
| --- | --- | --- | --- | --- | --- | --- |
|  | Facility-based service | Surveillance | Program Management | Outreach service | Social Mobilization | Supply Chain |
| (Intercept) | 6.73 *** | 11.58 *** | -1.53 | 5.18 *** | 6.14 * | 11.68 *** |
|  | (<0.001) | (<0.001) | (0.86) | (<0.001) | (0.01) | (<0.001) |
| Rural | -0.16 * | -0.64 * | 0.55 | -0.24 | -0.22 | -0.37 * |
|  | (0.01) | (0.02) | (0.51) | (0.07) | (0.34) | (<0.01) |
| Hospital | -0.41 * | -0.56 | -1.09 | -0.37 | -0.71 | 0.14 |
|  | (<0.01) | (0.38) | (0.6) | (0.24) | (0.20) | (0.65) |
| log(Number of doses) | 0.63 *** | -0.70 | 1.11 | 0.57 | 0.64 | -0.72 * |
|  | (<0.001) | (0.33) | (0.63) | (0.12) | (0.32) | (0.05) |
| log(Number of doses)^2^ | 0.02 | 0.08 | 0.03 | 0.02 | -0.01 | 0.07 * |
|  | (0.19) | (0.10) | (0.87) | (0.50) | (0.81) | (<0.01) |
| Adjusted R-squared | 0.97 | 0.44 | 0.30 | 0.88 | 0.42 | 0.59 |
| Correlation matrix for SUR regression residuals | | | | | | |
|  | Facility-based service | Surveillance | Program Management | Outreach service | Social Mobilization | Supply Chain |
| Facility-based service | 1.00 | 0.19 | 0.16 | 0.02 | 0.16 | 0.15 |
| Surveillance | 0.19 | 1.00 | 0.35 | 0.20 | 0.37 | 0.37 |
| Program Management | 0.16 | 0.35 | 1.00 | 0.25 | 0.18 | 0.11 |
| Outreach service | 0.02 | 0.20 | 0.25 | 1.00 | 0.06 | 0.07 |
| Social Mobilization | 0.16 | 0.37 | 0.18 | 0.06 | 1.00 | 0.08 |
| Supply Chain | 0.15 | 0.37 | 0.11 | 0.07 | 0.08 | 1.00 |
| McElroy R-squared (goodness of fit for system of equations) = 0.88. | | | | | | |
| *** Indicates statistical significance with p<0.001. * Indicates statistical significance with p<0.05. | | | | | | |

Figure S1. Changes in site-level cost shares across budget categories (without vaccine costs) related to increasing service delivery volume*.

*Bars within each country are displayed as increasing number of doses delivered, discretized by quintile. Percentages represent the weighted mean cost share of all facilities in each country.Table S15. Estimated value and relative difference of cost shares with 95% confidence intervals by budget category

| Comparison | Country | Value* | Estimated values and relative differences of cost shares by budget category | | | | | |
| --- | --- | --- | --- | --- | --- | --- | --- | --- |
|  |  |  | Labor | Vaccine | Cold Chain | Vehicle | Infrastructure | Per Diem |
| Hospital-based sites, compared to non-hospital-based sites | Uganda | Comparator | 31% (27, 35) | 49% (44, 53) | 6% (3, 11) | 6% (4, 11) | 7% (6, 9) | 1% (0,3) |
|  |  | Difference | 7% (-13, 21) | -23% (-36, -12) | -3% (-9, 3) | 12% (0, 31) | -2% (-5, 1) | 9% (-1, 53) |
|  | Benin | --- | --- | --- | --- | --- | --- | --- |
|  | Ghana | Comparator | 51% (10, 67) | 27% (5, 39) | 1% (0, 6) | 20% (0, 84) | 1% (0, 8) | 0% (0, 0) |
|  |  | Difference | 21% (-6, 60) | -1% (-22, 27) | 0% (-6, 4) | -18% (-79, 1) | -1% (-7, 0) | 0% (0, 0) |
|  | Zambia | Comparator | 45% (28, 52) | 31% (19, 36) | 1% (1, 2) | 12%(3, 46) | 4% (3, 5) | 6% (3, 8) |
|  |  | Difference | 7% (-9, 26) | -6% (-16, 8) | 1% (0, 2) | -11% (-44, -1) | 2% (-1, 5) | 8% (-2, 26) |
|  | Moldova | --- | --- | --- | --- | --- | --- | --- |
|  | Honduras | Comparator | 46% (43, 49) | 48% (45, 51) | 2% (2, 3) | --- | 3% (2, 3) | 2% (1, 3) |
|  |  | Difference | 8% (-11, 25) | -12% (-26, 4) | 1% (-1, 5) | --- | 1% (-2, 5) | 2% (-2, 16) |
| Non-rural-located sites, compared to rural-located sites | Uganda | Comparator | 30% (22, 37) | 44% (33, 51) | 5% (2, 11) | 10% (5, 19) | 7% (5, 9) | 4% (0, 26) |
|  |  | Difference | 6% (-4, 17) | 0% (-10, 11) | 0% (-7, 7) | -2% (-11, 6) | -1% (-4, 2) | -3% (-25, 4) |
|  | Benin | Comparator | 9% (7, 12) | 74% (69, 78) | 9% (7, 12) | 4% (2, 6) | 3% (2, 5) | 1% (0, 4) |
|  |  | Difference | 10% (5, 16) | -6% (-13, 1) | 0% (-4, 3) | -2% (-4, 0) | -2% (-3, 0) | 0% (-3, 0) |
|  | Ghana | Comparator | 54% (20, 67) | 30% (11, 41) | 2% (0, 5) | 14% (1, 69) | 1% (0, 3) | 0% (0, 0) |
|  |  | Difference | 13% (-1, 31) | -5% (-17, 9) | 0% (-3, 1) | -7% (-36, 8) | 0% (-2, 0) | 0% (0, 0) |
|  | Zambia | Comparator | 49% (28, 58) | 24% (14, 30) | 1% (1, 2) | 14% (3, 49) | 4% (2, 6) | 8% (4, 14) |
|  |  | Difference | -5% (-17, 16) | 18% (10, 30) | 1% (0, 2) | 1% (-1, 3) | 1% (-1, 3) | -2% (-9, 4) |
|  | Moldova | Comparator | 69% (61, 79) | 14% (9, 19) | 1% (1, 2) | 1% (1, 2) | 15% (11, 20) | 0% (0, 0) |
|  |  | Difference | 2% (-14, 17) | -2% (-10, 6) | 0% (-1, 1) | 0% (-9, 8) | 0% (-9, 8) | 0% (0, 0) |
|  | Honduras | Comparator | 44% (41, 48) | 49% (45, 52) | 2% (2, 3) | --- | 3% (3, 4) | 2% (1, 3) |
|  |  | Difference | 10% (2, 17) | -8% (-15, -2) | -1% (-1, 0) | --- | -1% (-2, 0) | 0% (-2, 1) |
| Non-government-owned sites, compared to government-owned sites | Uganda | Comparator | 36% (31, 41) | 47% (42, 51) | 5% (3, 10) | 5% (3, 9) | 5% (4, 6) | 2% (0, 8) |
|  |  | Difference | -9% (-20, 1) | -11% (-23, 0) | -1% (-7, 8) | 16% (4, 36) | 3% (-1, 8) | 1% (-4, 17) |
|  | Benin | Comparator | 13% (11, 15) | 72% (69, 75) | 9% (7, 11) | 3% (2, 5) | 2% (2, 3) | 0% (0, 2) |
|  |  | Difference | -8% (-12, -4) | 3% (-9, 12) | 3% (-3, 12) | -2% (-4, 0) | 5% (0, 15) | 0% (-2, 0) |
|  | Ghana | Comparator | 65% (58, 72) | 30% (24, 37) | 1% (0, 3) | 3% (1, 10) | 0% (0, 1) | 0% (0, 0) |
|  |  | Difference | -45% (-67, -8) | -11% (-33, 30) | 3% (-2, 27) | 48% (-2, 97) | 4% (0, 37) | 0% (0, 0) |
|  | Zambia | Comparator | 48% (39, 52) | 32% (26, 36) | 1% (1, 2) | 8% (2, 24) | 5% (4, 5) | 6% (4, 9) |
|  |  | Difference | -20% (-46, 6) | -13% (-31, 6) | 0% (-1, 3) | 24% (-11, 88) | 0% (-4, 6) | 9% (-6, 40) |
|  | Moldova | --- | --- | --- | --- | --- | --- | --- |
|  | Honduras | --- | --- | --- | --- | --- | --- | --- |
| Sites with service volume in the lowest quintile, compared to sites with one in the highest quintile | Uganda | Comparator | 22% (18, 27) | 60% (52, 66) | 8% (3, 17) | 6% (3, 11) | 4% (2, 5) | 1% (0, 4) |
|  |  | Difference | 26% (16, 35) | -31% (-40, -22) | -6% (-16, -1) | 3% (-4, 12) | 8% (4, 13) | 0% (-3, 6) |
|  | Benin | Comparator | 9% (6, 12) | 79% (74, 83) | 7% (5, 10) | 3% (1, 5) | 2% (1, 4) | 0% (0, 1) |
|  |  | Difference | 8% (2, 14) | -17% (-25, -9) | 5% (1, 10) | 3% (-1, 8) | 1% (-2, 3) | 0% (-1, 1) |
|  | Ghana | Comparator | 44% (15, 58) | 41% (13, 56) | 2% (0, 6) | 12% (1, 71) | 1% (0, 5) | 0% (0, 0) |
|  |  | Difference | 36% (4, 53) | -33% (-48, -8) | 0% (-4, 2) | -2% (-33, 26) | -1% (-5, 0) | 0% (0, 0) |
|  | Zambia | Comparator | 41% (20, 51) | 33% (15, 41) | 1% (0, 1) | 13% (1, 58) | 4% (2, 5) | 8% (4, 15) |
|  |  | Difference | 18% (-3, 37) | -19% (-29, -4) | 2% (0, 3) | 0% (-37, 30) | 2% (-1, 5) | -3% (-11, 3) |
|  | Moldova | Comparator | 72% (61, 82) | 14% (8, 22) | 0% (0, 1) | 1% (0, 2) | 12% (8, 18) | 0% (0, 0) |
|  |  | Difference | -20% (-33, -5) | -5% (-14, 1) | 7% (4, 9) | 2% (0, 4) | 17% (8, 25) | 0% (0, 0) |
|  | Honduras | Comparator | 32% (26, 40) | 65% (58, 72) | 1% (0, 1) | --- | 1% (0, 1) | 1% (0, 2) |
|  |  | Difference | 26% (17, 34) | -36% (-44, -27) | 3% (2, 5) | --- | 5% (4, 6) | 2% (0, 4) |

| Sites far from vaccine distribution center, compared to sites close to vaccine distribution center | Uganda | Comparator | 35% (29, 40) | 45% (40, 50) | 5% (2, 9) | 6% (4, 11) | 7% (5, 8) | 2% (0, 11) |
| --- | --- | --- | --- | --- | --- | --- | --- | --- |
|  |  | Difference | -1% (-5, 3) | -2% (-6, 2) | 1% (-2, 3) | 4% (1, 7) | 0% (-1, 1) | -1% (-6, 1) |
|  | Benin | Comparator | 14% (11, 16) | 73% (69, 76) | 9% (8, 12) | 2% (1, 3) | 2% (1, 3) | 1% (0, 2) |
|  |  | Difference | -1% (-3, 1) | 0% (-2, 3) | 0% (-2, 1) | 1% (0, 2) | 0% (0, 1) | 0% (-1, 0) |
|  | Ghana | Comparator | 60% (33, 70) | 31% (16, 39) | 1% (0, 2) | 8% (1, 50) | 0% (0, 1) | 0% (0, 0) |
|  |  | Difference | 2% (-3, 8) | -1% (-6, 4) | 0% (0, 1) | -1% (-11, 4) | 0% (0, 0) | 0% (0, 0) |
|  | Zambia | Comparator | 47% (29, 54) | 30% (19, 36) | 1% (1, 2) | 9% (1, 43) | 5% (3, 6) | 8% (4, 12) |
|  |  | Difference | -2% (-12, 9) | 1% (-6, 9) | 0% (0, 1) | 2% (-17, 20) | 0% (-2, 1) | -1% (-6, 3) |
|  | Moldova | Comparator | 70% (64, 75) | 13% (11, 16) | 1% (1, 2) | 1% (0, 1) | 15% (12, 18) | 0% (0, 0) |
|  |  | Difference | 2% (-3, 7) | -2% (-4, 1) | 0% (0, 0) | 0% (0, 1) | -1% (-4, 2) | 0% (0, 0) |
|  | Honduras | --- | --- | --- | --- | --- | --- | --- |

* ‘Comparator’ indicates the mean estimate for the comparator group (e.g. non-hospital based sites, for the first section of the table). ‘Difference’ indicates the mean difference between estimated values for the group of interest as compared to the comparator group (e.g. value for hospital based sites minus value for non-hospital based sites, for the first section of the table).

Table S16. Estimated value and relative difference of cost shares with 95% confidence intervals by programmatic activity

| Comparison | Country | Value* | Estimated values and relative differences of cost shares by programmatic activity | | | | | |
| --- | --- | --- | --- | --- | --- | --- | --- | --- |
|  |  |  | Facility-based service | Surveillance | Program Management | Outreach service | Social Mobilization | Supply Chain |
| Hospital-based sites, compared to non-hospital-based sites | Uganda | Comparator | 40% (23, 51) | 5% (2, 11) | 10% (6, 13) | 34% (17, 60) | 1% (1, 3) | 11% (6, 14) |
|  |  | Difference | -3% (-23, 17) | -2% (-8, 5) | 7% (0, 15) | -6% (-37, 27) | 0% (-2, 4) | 4% (-3, 12) |
|  | Benin | --- | --- | --- | --- | --- | --- | --- |
|  | Ghana | Comparator | 19% (10, 67) | 21% (5, 39) | 6%(0, 6) | 37% (1, 85) | 9% (0, 8) | 8% (4, 13) |
|  |  | Difference | 25% (-17, 69) | 1% (-20, 31) | 1% (-6, 14) | -21% (-56, 27) | -4% (-13, 7) | -1% (-9, 9) |
|  | Zambia | Comparator | 31% (19, 43) | 3% (2, 5) | 9% (5, 13) | 45%(25, 67) | 6% (3, 9) | 6% (4, 9) |
|  |  | Difference | 2% (-24, 27) | 0% (-3, 5) | 2% (-7, 13) | -12% (-50, 38) | 5% (-4, 23) | 3% (-4, 12) |
|  | Moldova | --- | --- | --- | --- | --- | --- | --- |
|  | Honduras | Comparator | 59% (49, 64) | 11% (9, 13) | 6% (1, 22) | 9% (7, 10) | 8% (6, 9) | 7% (6, 8) |
|  |  | Difference | -2% (-28, 16) | -1% (-9, 12) | 0% (-20, 36) | 0% (-5, 7) | -2% (-6, 6) | 5% (-1, 12) |
| Non-rural-located sites, compared to rural-located sites | Uganda | Comparator | 39% (21, 53) | 6% (2, 17) | 12% (7, 17) | 30% (12, 62) | 1% (0, 2) | 11% (6, 16) |
|  |  | Difference | 1% (-20, 21) | -3% (-14, 3) | -3% (-8, 3) | 4% (-28, 34) | 1% (-1, 6) | 0% (-6, 6) |
|  | Benin | Comparator | 55% (22, 73) | 3% (1, 4) | 2% (1, 3) | 29% (8, 27) | 2% (1, 3) | 10% (4, 14) |
|  |  | Difference | 14% (-5, 42) | 6% (3, 9) | 1% (-1, 3) | -23% (-59, -2) | 1% (-1, 2) | 3% (-3, 9) |
|  | Ghana | Comparator | 22% (8, 45) | 17% (9, 25) | 6% (3, 10) | 40% (20, 63) | 8% (4, 13) | 8% (4, 13) |
|  |  | Difference | 2% (-20, 24) | 17% (2, 32) | 1% (-4, 6) | -21% (-46, 4) | 3% (-4, 11) | -1% (-6, 4) |
|  | Zambia | Comparator | 27% (11, 42) | 4% (2, 8) | 9% (4, 15) | 47% (21, 78) | 5% (2, 10) | 7% (3, 11) |
|  |  | Difference | 8% (-18, 34) | -2% (-6, 1) | -1% (-9, 7) | -3% (-45, 40) | 1% (-6, 9) | -3% (-8, 2) |
|  | Moldova | Comparator | 52% (37, 61) | 10% (7, 12) | 21% (16, 29) | --- | 13% (7, 31) | 5% (3, 6) |
|  |  | Difference | -8% (-25, 10) | 8% (2, 13) | 3% (-7, 12) | --- | -2% (-20, 13) | 0% (-3, 4) |
|  | Honduras | Comparator | 60% 6048, 65) | 10% (8, 12) | 7% (2, 24) | 9% (7, 10) | 8% (6, 10) | 7% (6, 8) |
|  |  | Difference | -3% (--12, 7) | 5% (0, 12) | -3% (-16, 4) | 0% (-2, 3) | 0% (-3, 3) | 1% (-1, 3) |
| Non-government-owned sites, compared to government-owned sites | Uganda | Comparator | 41% (26, 52) | 4% (2, 9) | 10% (7, 13) | 32% (16, 55) | 2% (1, 4) | 11% (7, 14) |
|  |  | Difference | -3% (-24, 17) | 0% (-6, 10) | 1% (-5, 7) | 2% (-27, 36) | -1% (-4, 0) | 1% (-6, 8) |
|  | Benin | Comparator | 60% (39, 72) | 4% (3, 6) | 2% (1, 3) | 20% (6, 49) | 2% (1, 3) | 11% (7, 14) |
|  |  | Difference | 23% (7, 44) | -3% (-4, -1) | -1% (-2, 1) | -19% (-46, -5) | -1% (-2, 0) | 2% (-5, 11) |
|  | Ghana | Comparator | 26% (12, 48) | 23% (14, 32) | 7% (4, 11) | 30% (15, 49) | 8% (4, 14) | 7% (4, 10) |
|  |  | Difference | -11% (-44, 41) | -16% (-29, 3) | -5% (-10, 3) | 18% (-34, 70) | 4% (-9, 37) | 10% (-6, 42) |
|  | Zambia | Comparator | 31% (18, 42) | 3% (2, 5) | 9% (5, 13) | 45% (26, 66) | 6% (3, 9) | 6% (4, 9) |
|  |  | Difference | 4% (-28, 37) | 0% (-3, 7) | 4% (-8, 21) | -9% (-53, 50) | 2% (-6, 24) | -1% (-6, 8) |
|  | Moldova | --- | --- | --- | --- | --- | --- | --- |
|  | Honduras | --- | --- | --- | --- | --- | --- | --- |
| Sites with service volume in the lowest quintile, compared to sites with one in the highest | Uganda | Comparator | 38% (20, 55) | 4% (1, 13) | 6% (4, 9) | 43% (21, 70) | 1% (0, 2) | 7% (4, 11) |
|  |  | Difference | 5% (-16, 26) | 1% (-8, 9) | 13% (8, 18) | -32% (-61, -5) | 2% (-1, 6) | 12% (6, 18) |
|  | Benin | Comparator | 64% (30, 82) | 2% (1, 4) | 2% (1, 4) | 22% (3, 64) | 1% (1, 2) | 8% (4, 12) |
|  |  | Difference | -12% (-41, 25) | 3% (0, 6) | 0% (-3, 2) | 0% (-44, 38) | 1% (0, 3) | 7% (0, 14) |
|  | Ghana | Comparator | 33% (13, 58) | 15% (7, 24) | 8% (4, 14) | 30% (10, 60) | 8% (3, 15) | 7% (3, 13) |
|  |  | Difference | -28% (-54, -7) | 28% (8, 47) | -4% (-11, 2) | -6% (-40, 28) | 10% (-1, 24) | 0% (-7, 6) |
|  | Zambia | Comparator | 20% (5, 38) | 2% (1, 5) | 5% (1, 10) | 66% (35, 91) | 3% (1, 7) | 4% (1, 7) |
|  |  | Difference | 20% (-2, 39) | 2% (-1, 6) | 13% (5, 21) | -58% (-86, -22) | 10% (1, 22) | 12% (6, 17) |
|  | Moldova | Comparator | 49% (37, 61) | 10% (8, 14) | 24% (17, 32) | --- | 13% (5, 28) | 3% (2, 5) |
|  |  | Difference | 10% (-5, 25) | -1% (-5, 3) | -10% (-18, -2) | --- | -8% (-22, 2) | 9% (5, 12) |
|  | Honduras | Comparator | 65% (43, 76) | 7% (4, 10) | 13% (2, 41) | 9% (6, 12) | 4% (2, 6) | 2% (2, 3) |
|  |  | Difference | -15% (-28, 8) | 10% (4, 15) | -12% (-40, -1) | -1% (-5, 2) | 8% (5, 12) | 11% (9, 13) |

| Sites far from vaccine distribution center, compared to sites close to vaccine distribution center | Uganda | Comparator | 41% (27, 52) | 4% (2, 9) | 11% (7, 14) | 31% (16, 54) | 1% (0, 3) | 11% (7, 14) |
| --- | --- | --- | --- | --- | --- | --- | --- | --- |
|  |  | Difference | -2% (-10, 6) | 0% (-3, 2) | -1% (-3, 2) | 2% (-10, 14) | 0% (-1, 1) | 0% (-2, 3) |
|  | Benin | Comparator | 68% (49, 77) | 4% (3, 6) | 2% (1, 3) | 13% (3, 36) | 2% (1, 2) | 12% (8, 14) |
|  |  | Difference | -5% (-15, 3) | 0% (-1, 0) | 0% (0, 1) | 5% (-5, 18) | 0% (0, 1) | 0% (-2, 2) |
|  | Ghana | Comparator | 25% (11, 44) | 23% (15, 33) | 6% (4, 9) | 31% (16, 52) | 7% (4, 12) | 8% (5, 12) |
|  |  | Difference | 0% (-10, 9) | 0% (-7, 5) | 0% (-2, 2) | -1% (-13, 11) | 2% (-1, 4) | 0% (-3, 2) |
|  | Zambia | Comparator | 32% (18, 45) | 4% (2, 6) | 10% (6, 15) | 41% (21, 66) | 6% (3, 11) | 6% (4, 9) |
|  |  | Difference | -6% (-24, 12) | -1% (-4, 1) | -4% (-9, 2) | 12% (-19, 43) | -1% (-6, 5) | 0% (-4, 4) |
|  | Moldova | Comparator | 49% (43, 54) | 13% (11, 15) | 23% (19, 27) | --- | 12% (8, 17) | 4% (3, 5) |
|  |  | Difference | -2% (-7, 4) | 2% (0, 4) | 2% (-1, 5) | --- | -2% (-6, 2) | 1% (0, 2) |
|  | Honduras | --- | --- | --- | --- | --- | --- | --- |

* ‘Comparator’ indicates the mean estimate for the comparator group (e.g. non-hospital based sites, for the first section of the table). ‘Difference’ indicates the mean difference between estimated values for the group of interest as compared to the comparator group (e.g. value for hospital based sites minus value for non-hospital based sites, for the first section of the table).

Table S17. Coefficient estimates for regression analysis of relationship between site characteristics and total costs of each site for each country.

| Coefficient estimates for SUR regression equations (coefficient, (standard error)) | | | | | | |
| --- | --- | --- | --- | --- | --- | --- |
|  | Uganda | Benin | Ghana | Zambia | Moldova | Honduras |
| (Intercept) | 14.6 *** | 21.13 *** | 18.53 *** | 13.93 | 6.10 | 9.28 |
|  | (<0.001) | (<0.001) | (<0.001) | (<0.001) | (<0.001) | (<0.001) |
| Rural | -0.04 | -0.16 | -0.22 | 0.58 | -0.16 | -0.21 |
|  | 0.68 | 0.06 | 0.15 | (<0.001) | 0.62 | 0.01 |
| Hospital | 0.49 *** | --- | -0.33 | 0.35 | --- | -0.38 |
|  | (<0.001) | --- | 0.26 | 0.04 | --- | 0.06 |
| Government-owned | -0.22 * | 0.09 | -0.63 | -0.11 | --- | --- |
|  | 0.04 | 0.50 | 0.09 | 0.65 | --- | --- |
| Distance | 0.01 | 0.00 | 0.01 | 0.00 | 0.00 | --- |
|  | 0.16 | 0.95 | 0.05 | 0.25 | 0.34 | --- |
| log(Number of doses) | -0.06 | -1.85 | -2.46 * | 0.47 | 0.82 | 0.23 |
|  | 0.85 | 0.14 | (<0.01) | 0.44 | 0.01 | 0.31 |
| log(Number of doses)^2^ | 0.04 * | 0.14 | 0.19 * | 0.01 | 0.00 | 0.03 |
|  | 0.02 | 0.06 | (<0.01) | 0.82 | 0.89 | 0.03 |
| Adjusted R-squared | 0.91 | 0.81 | 0.47 | 0.75 | 0.90 | 0.93 |

*** Indicates statistical significance with p<0.001. * Indicates statistical significance with p<0.05.

Table S18. Coefficient estimates and residual correlation for regression analysis of relationship between residuals of total costs regression (efficiency index) and cost shares for each budget category (Uganda).

| Coefficient estimates for SUR regression equations (coefficient, (standard error)) | | | | | | |
| --- | --- | --- | --- | --- | --- | --- |
|  | Labor | Vaccine | Cold Chain | Vehicle | Infrastructure | Per Diem |
| (Intercept) | 15.94 *** | 16.26 *** | 14.06 *** | 14.22 *** | 14.11 *** | 11.05*** |
|  | (<0.001) | (<0.001) | (<0.001) | (<0.001) | (<0.001) | (<0.001) |
| Residual | 1.67 * | 0.52 | 1.56 | 3.33 * | 0.38 | 0.39 |
|  | (<0.01) | 0.48 | 0.31 | (<0.01) | 0.42 | 0.89 |
| (Residual)^2^ | -1.16 | -3.62 | -8.66 | -3.23 | -1.18 | -2.09 |
|  | 0.54 | 0.17 | 0.12 | 0.42 | 0.49 | 0.84 |
| Adjusted R-squared | 0.16 | 0.00 | 0.01 | 0.14 | -0.03 | -0.04 |
| Correlation matrix for SUR regression residuals | | | | | | |
|  | Labor | Vaccine | Cold Chain | Vehicle | Infrastructure | Per Diem |
| Labor | 1.00 | 0.83 | 0.39 | 0.50 | 0.17 | 0.18 |
| Vaccine | 0.83 | 1.00 | 0.53 | 0.52 | 0.38 | 0.20 |
| Cold Chain | 0.39 | 0.53 | 1.00 | 0.36 | 0.39 | 0.39 |
| Vehicle | 0.50 | 0.52 | 0.36 | 1.00 | 0.34 | 0.28 |
| Infrastructure | 0.17 | 0.38 | 0.39 | 0.34 | 1.00 | 0.22 |
| Per Diem | 0.18 | 0.20 | 0.39 | 0.28 | 0.22 | 1.00 |
| McElroy R-squared (goodness of fit for system of equations) = 0.15. | | | | | | |
| *** Indicates statistical significance with p<0.001. * Indicates statistical significance with p<0.05. | | | | | | |

Table S19. Coefficient estimates and residual correlation for regression analysis of relationship between residuals of total costs regression (efficiency index) and cost shares for each budget category (Benin).

| Coefficient estimates for SUR regression equations (coefficient, (standard error)) | | | | | | |
| --- | --- | --- | --- | --- | --- | --- |
|  | Labor | Vaccine | Cold Chain | Vehicle | Infrastructure | Per Diem |
| (Intercept) | 13.90 *** | 15.55 *** | 13.59 *** | 11.84 *** | 12.27 *** | 11.05 *** |
|  | (<0.001) | (<0.001) | (<0.001) | (<0.001) | (<0.001) | (<0.001) |
| Residual | 0.99 | 1.16 * | 1.10 * | -0.11 | 1.43 | 0.39 |
|  | 0.16 | 0.02 | 0.04 | 0.93 | 0.09 | 0.89 |
| (Residual)^2^ | -1.63 | -0.68 | -1.30 | 2.74 | -4.19 | -2.09 |
|  | 0.39 | 0.62 | 0.37 | 0.39 | 0.07 | 0.84 |
| Adjusted R-squared | 0.00 | 0.09 | 0.06 | -0.03 | 0.06 | -0.04 |
| Correlation matrix for SUR regression residuals | | | | | | |
|  | Labor | Vaccine | Cold Chain | Vehicle | Infrastructure | Per Diem |
| Labor | 1.00 | 0.44 | 0.30 | -0.17 | -0.24 | 0.13 |
| Vaccine | 0.44 | 1.00 | 0.28 | 0.13 | 0.06 | 0.02 |
| Cold Chain | 0.30 | 0.28 | 1.00 | -0.26 | -0.15 | -0.19 |
| Vehicle | -0.17 | 0.13 | -0.26 | 1.00 | 0.41 | 0.46 |
| Infrastructure | -0.24 | 0.06 | -0.15 | 0.41 | 1.00 | 0.34 |
| Per Diem | 0.13 | 0.02 | -0.19 | 0.46 | 0.34 | 1.00 |
| McElroy R-squared (goodness of fit for system of equations) = 0.08. | | | | | | |
| *** Indicates statistical significance with p<0.001. * Indicates statistical significance with p<0.05. | | | | | | |

Table S20. Coefficient estimates and residual correlation for regression analysis of relationship between residuals of total costs regression (efficiency index) and cost shares for each budget category (Ghana).

| Coefficient estimates for SUR regression equations (coefficient, (standard error)) | | | | | | |
| --- | --- | --- | --- | --- | --- | --- |
|  | Labor | Vaccine | Cold Chain | Vehicle | Infrastructure | Per Diem |
| (Intercept) | 9.67 *** | 8.55 *** | 4.52 *** | 5.99 *** | 2.99 *** | 2.48 |
|  | (<0.001) | (<0.001) | (<0.001) | (<0.001) | (<0.001) | (<0.001) |
| Residual | 1.17 *** | 0.69 | 0.36 | 1.46 | 1.90 | -0.22 |
|  | (<0.001) | 0.09 | 0.67 | 0.20 | 0.07 | 0.74 |
| (Residual)^2^ | -0.41 | 0.25 | 1.18 | -2.07 | -0.58 | 0.44 |
|  | 0.15 | 0.69 | 0.35 | 0.23 | 0.71 | 0.66 |
| Adjusted R-squared | 0.45 | 0.05 | 0.00 | 0.00 | 0.03 | -0.04 |
| Correlation matrix for SUR regression residuals | | | | | | |
|  | Labor | Vaccine | Cold Chain | Vehicle | Infrastructure | Per Diem |
| Labor | 1.00 | 0.19 | 0.11 | -0.12 | 0.04 | 0.23 |
| Vaccine | 0.19 | 1.00 | 0.14 | -0.10 | 0.33 | 0.12 |
| Cold Chain | 0.11 | 0.14 | 1.00 | -0.08 | 0.48 | 0.12 |
| Vehicle | -0.12 | -0.10 | -0.08 | 1.00 | 0.16 | 0.18 |
| Infrastructure | 0.04 | 0.33 | 0.48 | 0.16 | 1.00 | 0.10 |
| Per Diem | 0.23 | 0.12 | 0.12 | 0.18 | 0.10 | 1.00 |
| McElroy R-squared (goodness of fit for system of equations) = 0.17. | | | | | | |
| *** Indicates statistical significance with p<0.001. * Indicates statistical significance with p<0.05. | | | | | | |

Table S21. Coefficient estimates and residual correlation for regression analysis of relationship between residuals of total costs regression (efficiency index) and cost shares for each budget category (Zambia).

| Coefficient estimates for SUR regression equations (coefficient, (standard error)) | | | | | | |
| --- | --- | --- | --- | --- | --- | --- |
|  | Labor | Vaccine | Cold Chain | Vehicle | Infrastructure | Per Diem |
| (Intercept) | 18.00 *** | 17.27 *** | 14.51 *** | 14.74 *** | 15.65 *** | 16.05 *** |
|  | (<0.001) | (<0.001) | (<0.001) | (<0.001) | (<0.001) | (<0.001) |
| Residual | 1.22 *** | 0.16 | 0.05 | 2.11 | 0.46 | 2.15 |
|  | (<0.001) | 0.74 | 0.88 | 0.21 | 0.11 | (<0.001) |
| (Residual)^2^ | -0.93 | -0.32 * | -0.95 | 1.09 | -0.87 | -3.77 |
|  | 0.14 | 0.01 | 0.26 | 0.81 | 0.25 | 0.01 |
| Adjusted R-squared | 0.35 | 0.09 | -0.01 | 0.00 | 0.03 | 0.27 |
| Correlation matrix for SUR regression residuals | | | | | | |
|  | Labor | Vaccine | Cold Chain | Vehicle | Infrastructure | Per Diem |
| Labor | 1.00 | 0.64 | 0.16 | 0.03 | 0.42 | 0.23 |
| Vaccine | 0.64 | 1.00 | 0.19 | -0.16 | 0.59 | 0.34 |
| Cold Chain | 0.16 | 0.19 | 1.00 | -0.17 | 0.36 | 0.25 |
| Vehicle | 0.03 | -0.16 | -0.17 | 1.00 | -0.22 | 0.03 |
| Infrastructure | 0.42 | 0.59 | 0.36 | -0.22 | 1.00 | 0.43 |
| Per Diem | 0.23 | 0.34 | 0.25 | 0.03 | 0.43 | 1.00 |
| McElroy R-squared (goodness of fit for system of equations) = 0.2. | | | | | | |
| *** Indicates statistical significance with p<0.001. * Indicates statistical significance with p<0.05. | | | | | | |

Table S22. Coefficient estimates and residual correlation for regression analysis of relationship between residuals of total costs regression (efficiency index) and cost shares for each budget category (Moldova).

| Coefficient estimates for SUR regression equations (coefficient, (standard error)) | | | | | | |
| --- | --- | --- | --- | --- | --- | --- |
|  | Labor | Vaccine | Cold Chain | Vehicle | Infrastructure | Per Diem |
| (Intercept) | 10.14 *** | 8.33 *** | 6.42 *** | 6.05 *** | 8.72 *** | 1.74 *** |
|  | (<0.001) | (<0.001) | (<0.001) | (<0.001) | (<0.001) | 0.02 |
| Residual | 1.19 * | -0.03 | -0.06 | 0.53 | 0.8 * | 0.99 |
|  | 0.02 | 0.96 | 0.75 | 0.27 | 0.05 | 0.57 |
| (Residual)^2^ | -1.33 | -1.03 | 0.07 | -0.10 | -0.67 | 0.70 |
|  | 0.12 | 0.24 | 0.82 | 0.91 | 0.32 | 0.03 |
| Adjusted R-squared | 0.14 | -0.01 | -0.04 | -0.01 | 0.08 | -0.01 |
| Correlation matrix for SUR regression residuals | | | | | | |
|  | Labor | Vaccine | Cold Chain | Vehicle | Infrastructure | Per Diem |
| Labor | 1.00 | 0.98 | 0.53 | 0.59 | 0.90 | 0.26 |
| Vaccine | 0.98 | 1.00 | 0.56 | 0.57 | 0.94 | 0.26 |
| Cold Chain | 0.53 | 0.56 | 1.00 | 0.23 | 0.49 | 0.13 |
| Vehicle | 0.59 | 0.57 | 0.23 | 1.00 | 0.55 | 0.34 |
| Infrastructure | 0.90 | 0.94 | 0.49 | 0.55 | 1.00 | 0.26 |
| Per Diem | 0.26 | 0.26 | 0.13 | 0.34 | 0.26 | 1.00 |
| McElroy R-squared (goodness of fit for system of equations) = 0.62. | | | | | | |
| *** Indicates statistical significance with p<0.001. * Indicates statistical significance with p<0.05. | | | | | | |

Table S23. Coefficient estimates and residual correlation for regression analysis of relationship between residuals of total costs regression (efficiency index) and cost shares for each budget category (Honduras).

| Coefficient estimates for SUR regression equations (coefficient, (standard error)) | | | | | | |
| --- | --- | --- | --- | --- | --- | --- |
|  | Labor | Vaccine | Cold Chain | Infrastructure | Per Diem | |
| (Intercept) | 12.00 *** | 11.85 *** | 8.67 *** | 9.01 *** | 8.57 *** | |
|  | (<0.001) | (<0.001) | (<0.001) | (<0.001) | (<0.001) | |
| Residual | 2.13 *** | 0.61 | 0.08 | 0.26 | 2.82 *** | |
|  | (<0.001) | 0.35 | 0.81 | 0.39 | (<0.001) | |
| (Residual)^2^ | -1.96 * | -2.10 | -0.41 | -0.83 | -3.02 | |
|  | 0.02 | 0.10 | 0.56 | 0.16 | 0.06 | |
| Adjusted R-squared | 0.24 | 0.01 | -0.02 | 0.00 | 0.14 | |
| Correlation matrix for SUR regression residuals | | | | | | |
|  | Labor | Vaccine | Cold Chain | Infrastructure | Per Diem | |
| Labor | 1.00 | 0.94 | 0.36 | 0.38 | 0.32 | |
| Vaccine | 0.94 | 1.00 | 0.48 | 0.51 | 0.32 | |
| Cold Chain | 0.36 | 0.48 | 1.00 | 0.41 | 0.21 | |
| Infrastructure | 0.38 | 0.51 | 0.41 | 1.00 | 0.18 | |
| Per Diem | 0.32 | 0.32 | 0.21 | 0.18 | 1.00 | |
| McElroy R-squared (goodness of fit for system of equations) = 0.34. | | | |  | |  |
| *** Indicates statistical significance with p<0.001. * Indicates statistical significance with p<0.05. | | | | | |  |

Table S24. Coefficient estimates and residual correlation for regression analysis of relationship between residuals of total costs regression (efficiency index) and cost shares for each programmatic activity (Uganda).

| Coefficient estimates for SUR regression equations (coefficient, (standard error)) | | | | | | |
| --- | --- | --- | --- | --- | --- | --- |
|  | Facility-based service | Surveillance | Program Management | Outreach service | Social Mobilization | Supply Chain |
| (Intercept) | 16.26 *** | 13.67 *** | 15.02 *** | 15.94 *** | 12.52 *** | 14.93 *** |
|  | (<0.001) | (<0.001) | (<0.001) | (<0.001) | (<0.001) | (<0.001) |
| Residual | 0.86 | 3.03 * | 2.14 *** | 3.48 * | 2.78 | 1.43 |
|  | 0.17 | 0.03 | (<0.001) | 0.03 | 0.04 * | (<0.001) |
| (Residual)^2^ | -2.37 | -3.68 | -2.71 | -8.57 | -0.71 | -0.78 |
|  | 0.30 | 0.45 | 0.13 | 0.13 | 0.88 | 0.68 |
| Adjusted R-squared | 0.00 | 0.06 | 0.27 | 0.07 | 0.07 | 0.12 |
| Correlation matrix for SUR regression residuals | | | | | | |
|  | Facility-based service | Surveillance | Program Management | Outreach service | Social Mobilization | Supply Chain |
| Facility-based service | 1.00 | 0.17 | 0.71 | 0.55 | 0.31 | 0.79 |
| Surveillance | 0.17 | 1.00 | 0.27 | 0.13 | 0.13 | 0.18 |
| Program Management | 0.71 | 0.27 | 1.00 | 0.49 | 0.35 | 0.76 |
| Outreach service | 0.55 | 0.13 | 0.49 | 1.00 | 0.25 | 0.44 |
| Social Mobilization | 0.31 | 0.13 | 0.35 | 0.25 | 1.00 | 0.32 |
| Supply Chain | 0.79 | 0.18 | 0.76 | 0.44 | 0.32 | 1.00 |
| McElroy R-squared (goodness of fit for system of equations) = 0.11. | | | | | | |
| *** Indicates statistical significance with p<0.001. * Indicates statistical significance with p<0.05. | | | | | | |

Table S25. Coefficient estimates and residual correlation for regression analysis of relationship between residuals of total costs regression (efficiency index) and cost shares for each programmatic activity (Benin).

| Coefficient estimates for SUR regression equations (coefficient, (standard error)) | | | | | | |
| --- | --- | --- | --- | --- | --- | --- |
|  | Facility-based service | Surveillance | Program Management | Outreach service | Social Mobilization | Supply Chain |
| (Intercept) | 15.45 *** | 12.74 *** | 11.76 *** | 13.10 *** | 11.90 *** | 13.83 *** |
|  | (<0.001) | (<0.001) | (<0.001) | (<0.001) | (<0.001) | (<0.001) |
| Residual | 1.08 * | 1.21 | 0.54 | 5.58 * | -0.34 | 1.08 * |
|  | 0.04 | 0.14 | 0.64 | 0.05 | 0.70 | 0.02 |
| (Residual)^2^ | -0.80 | -1.66 | -0.42 | -7.33 | -0.68 | -1.17 |
|  | 0.58 | 0.45 | 0.89 | 0.34 | 0.78 | 0.36 |
| Adjusted R-squared | 0.05 | 0.01 | -0.04 | 0.05 | -0.03 | 0.08 |
| Correlation matrix for SUR regression residuals | | | | | | |
|  | Facility-based service | Surveillance | Program Management | Outreach service | Social Mobilization | Supply Chain |
| Facility-based service | 1.00 | 0.50 | 0.31 | -0.18 | 0.19 | 0.29 |
| Surveillance | 0.50 | 1.00 | 0.23 | -0.07 | 0.21 | 0.38 |
| Program Management | 0.31 | 0.23 | 1.00 | 0.27 | 0.43 | 0.26 |
| Outreach service | -0.18 | -0.07 | 0.27 | 1.00 | 0.35 | -0.03 |
| Social Mobilization | 0.19 | 0.21 | 0.43 | 0.35 | 1.00 | 0.01 |
| Supply Chain | 0.29 | 0.38 | 0.26 | -0.03 | 0.01 | 1.00 |
| McElroy R-squared (goodness of fit for system of equations) = 0.07. | | | | | | |
| *** Indicates statistical significance with p<0.001. * Indicates statistical significance with p<0.05. | | | | | | |

Table S26. Coefficient estimates and residual correlation for regression analysis of relationship between residuals of t

otal costs regression (efficiency index) and cost shares for each programmatic activity (Ghana).

| Coefficient estimates for SUR regression equations (coefficient, (standard error)) | | | | | | |
| --- | --- | --- | --- | --- | --- | --- |
|  | Facility-based service | Surveillance | Program Management | Outreach service | Social Mobilization | Supply Chain |
| (Intercept) | 7.72 *** | 8.43 *** | 7.15 *** | 8.26 *** | 7.48 *** | 7.35 |
|  | (<0.001) | (<0.001) | (<0.001) | (<0.001) | (<0.001) | (<0.001) |
| Residual | 1.11 | 1.09 *** | 1.34 | 0.47 | 1.75 | 1.02 |
|  | 0.16 | (<0.001) | (<0.01) | 0.51 | (<0.001) | 0.01 * |
| (Residual)^2^ | 0.97 | -0.16 | -0.47 | 0.72 | -1.48 | -0.47 |
|  | 0.41 | 0.71 | 0.49 | 0.49 | 0.02 | 0.42 |
| Adjusted R-squared | 0.05 | 0.23 | 0.13 | -0.01 | 0.24 | 0.09 |
| Correlation matrix for SUR regression residuals | | | | | | |
|  | Facility-based service | Surveillance | Program Management | Outreach service | Social Mobilization | Supply Chain |
| Facility-based service | 1.00 | -0.13 | 0.43 | -0.13 | 0.01 | 0.36 |
| Surveillance | -0.13 | 1.00 | 0.26 | -0.37 | 0.08 | -0.31 |
| Program Management | 0.43 | 0.26 | 1.00 | -0.14 | 0.04 | 0.28 |
| Outreach service | -0.13 | -0.37 | -0.14 | 1.00 | -0.03 | 0.12 |
| Social Mobilization | 0.01 | 0.08 | 0.04 | -0.03 | 1.00 | 0.07 |
| Supply Chain | 0.36 | -0.31 | 0.28 | 0.12 | 0.07 | 1.00 |
| McElroy R-squared (goodness of fit for system of equations) = 0.18. | | | | | | |
| *** Indicates statistical significance with p<0.001. * Indicates statistical significance with p<0.05. | | | | | | |

Table S27. Coefficient estimates and residual correlation for regression analysis of relationship between residuals of total costs regression (efficiency index) and cost shares for each programmatic activity (Zambia).

| Coefficient estimates for SUR regression equations (coefficient, (standard error)) | | | | | | | | | | | | |
| --- | --- | --- | --- | --- | --- | --- | --- | --- | --- | --- | --- | --- |
|  | | Facility-based service | | Surveillance | | Program Management | | Outreach service | | Social Mobilization | | Supply Chain |
| (Intercept) | | 17.44 *** | | 15.31 *** | | 16.43 *** | | 17.62 *** | | 16.11 *** | | 16.05 *** |
|  |  | (<0.001) | | (<0.001) | | (<0.001) | | (<0.001) | | (<0.001) | | (<0.001) |
| Residual | | 0.43 | | 2.19 | | 1.24 | | 3.27 * | | 1.35 * | | 1.03 |
|  |  | 0.26 | | (<0.001) | | (<0.001) | | (<0.01) | | 0.01 | | (<0.01) |
| (Residual)^2^ | | -1.16 | | -0.38 | | -0.85 | | -6.55 * | | -3.46 * | | 0.27 |
|  |  | 0.26 | | 0.73 | | 0.30 | | 0.04 | | 0.02 | | 0.76 |
| Adjusted R-squared | | 0.01 | | 0.34 | | 0.24 | | 0.15 | | 0.03 | | 0.13 |
| Correlation matrix for SUR regression residuals | | | | | | | | | | | | |
|  | Facility-based service | | Surveillance | | Program Management | | Outreach service | | Social Mobilization | | Supply Chain | |
| Facility-based service | 1.00 | | 0.50 | | 0.40 | | 0.26 | | 0.08 | | 0.02 | |
| Surveillance | 0.50 | | 1.00 | | 0.52 | | 0.05 | | -0.01 | | 0.39 | |
| Program Management | 0.40 | | 0.52 | | 1.00 | | -0.03 | | -0.18 | | 0.31 | |
| Outreach service | 0.26 | | 0.05 | | -0.03 | | 1.00 | | -0.01 | | 0.08 | |
| Social Mobilization | 0.08 | | -0.01 | | -0.18 | | -0.01 | | 1.00 | | -0.06 | |
| Supply Chain | 0.02 | | 0.39 | | 0.31 | | 0.08 | | -0.06 | | 1.00 | |
| McElroy R-squared (goodness of fit for system of equations) = 0.2. | | | | | | | | | | | | |
| *** Indicates statistical significance with p<0.001. * Indicates statistical significance with p<0.05. | | | | | | | | | | | | |

Table S28. Coefficient estimates and residual correlation for regression analysis of relationship between residuals of total costs regression (efficiency index) and cost shares for each programmatic activity (Moldova).

| Coefficient estimates for SUR regression equations (coefficient, (standard error)) | | | | | | | |
| --- | --- | --- | --- | --- | --- | --- | --- |
|  | Facility-based service | Surveillance | Program Management | Social Mobilization | | Supply Chain | |
| (Intercept) | 9.87 *** | 8.35 *** | 8.92 *** | 8.18 *** | | 7.67 *** | |
|  | (<0.001) | (<0.001) | (<0.001) | (<0.001) | | (<0.001) | |
| Residual | 0.66 | 1.04 | 1.26 * | 1.65 * | | .86 *** | |
|  | 0.14 | 0.05 | 0.02 | 0.01 | | 0.01 | |
| (Residual)^2^ | -0.90 | -1.17 | -1.42 | -1.65 | | -0.07 | |
|  | 0.22 | 0.19 | 0.12 | 0.13 | | 0.90 | |
| Adjusted R-squared | 0.06 | 0.10 | 0.14 | 0.16 | | 0.10 | |
| Correlation matrix for SUR regression residuals | | | | | | | |
|  | Facility-based service | Surveillance | Program Management | Social Mobilization | | Supply Chain | |
| Facility-based service | 1.00 | 0.95 | 0.97 | 0.85 | | 0.84 | |
| Surveillance | 0.95 | 1.00 | 0.97 | 0.87 | | 0.88 | |
| Program Management | 0.97 | 0.97 | 1.00 | 0.85 | | 0.89 | |
| Social Mobilization | 0.85 | 0.87 | 0.85 | 1.00 | | 0.77 | |
| Supply Chain | 0.84 | 0.88 | 0.89 | 0.77 | | 1.00 | |
| McElroy R-squared (goodness of fit for system of equations) = 0.15. | | | | |  | |  |
| *** Indicates statistical significance with p<0.001. * Indicates statistical significance with p<0.05. | | | | | | |  |

Table S29. Coefficient estimates and residual correlation for regression analysis of relationship between residuals of total costs regression (efficiency index) and cost shares for each programmatic activity (Honduras).

| Coefficient estimates for SUR regression equations (coefficient, (standard error)) | | | | | | |
| --- | --- | --- | --- | --- | --- | --- |
|  | Facility-based service | Surveillance | Program Management | Outreach service | Social Mobilization | Supply Chain |
| (Intercept) | 12.17 *** | 10.50 *** | 8.76 *** | 10.25 *** | 10.06 *** | 10.50 *** |
|  | (<0.001) | (<0.001) | (<0.001) | (<0.001) | (<0.001) | (<0.001) |
| Residual | 0.89 | 2.50 | 5.92 | 0.98 | 1.62 *** | 2.50 |
|  | 0.13 | (<0.001) | (<0.001) | 0.10 | (<0.001) | (<0.001) |
| (Residual)^2^ | -2.23 | -1.53 | -4.98 | -2.06 | -0.22 | -1.53 |
|  | 0.05 | 0.11 | 0.09 | 0.08 | 0.80 | 0.11 |
| Adjusted R-squared | 0.04 | 0.26 | 0.16 | 0.03 | 0.15 | 0.26 |
| Correlation matrix for SUR regression residuals | | | | | | |
|  | Facility-based service | Surveillance | Program Management | Outreach service | Social Mobilization | Supply Chain |
| Facility-based service | 1.00 | 0.70 | 0.58 | 0.92 | 0.70 | 0.72 |
| Surveillance | 0.70 | 1.00 | 0.44 | 0.69 | 0.54 | 0.67 |
| Program Management | 0.58 | 0.44 | 1.00 | 0.60 | 0.38 | 0.37 |
| Outreach service | 0.92 | 0.69 | 0.60 | 1.00 | 0.65 | 0.68 |
| Social Mobilization | 0.70 | 0.54 | 0.38 | 0.65 | 1.00 | 0.47 |
| Supply Chain | 0.72 | 0.67 | 0.37 | 0.68 | 0.47 | 1.00 |
| McElroy R-squared (goodness of fit for system of equations) = 0.16. | | | | | | |
| *** Indicates statistical significance with p<0.001. * Indicates statistical significance with p<0.05. | | | | | | |

Figure S2. Changes in the distribution of total costs (Panel A), and the cost per dose (Panel B), across programmatic activity categories related to increasing efficiency index.*


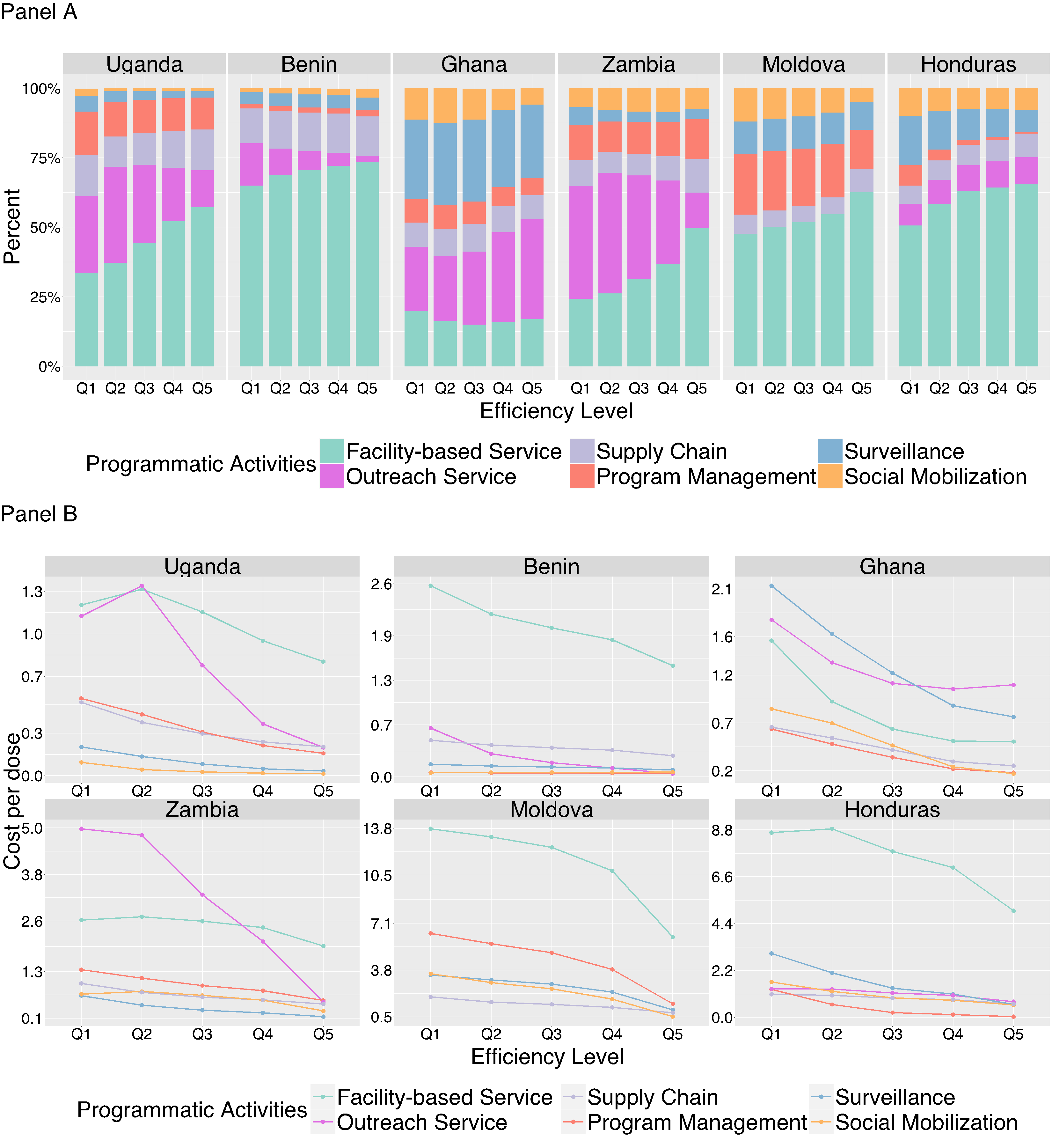


* Bars within each country are displayed as increasing number of doses delivered, discretized by quintile. Percentages represent the weighted mean cost share of all facilities in each country.

Table S30. Statistical testing results for differences in the cost shares estimated for the sites with lowest and highest quintiles of service volume, across budget category.

| Country | P value of two-tailed t-test for estimated differences of cost share by budget category | | | | | |
| --- | --- | --- | --- | --- | --- | --- |
|  | Labor | Vaccine | Cold Chain | Vehicle | Infrastructure | Per Diem |
| Uganda | <0.001 | <0.001 | <0.001 | <0.001 | <0.001 | <0.001 |
| Benin | <0.001 | <0.001 | <0.001 | <0.001 | <0.001 | <0.001 |
| Ghana | <0.001 | <0.001 | <0.001 | <0.001 | <0.001 | <0.001 |
| Zambia | <0.001 | <0.001 | <0.001 | 0.040 | <0.001 | <0.001 |
| Moldova | <0.001 | <0.001 | <0.001 | <0.001 | <0.001 | <0.001 |
| Honduras | <0.001 | <0.001 | <0.001 | --- | <0.001 | <0.001 |

Table S31. Statistical testing results for differences in the cost shares estimated for the sites with lowest and highest quintiles of service volume, across programmatic activity.

| Country | P value of two-tailed t-test for estimated differences of cost share by programmatic activity | | | | | |
| --- | --- | --- | --- | --- | --- | --- |
|  | Facility-based service | Surveillance | Program Management | Outreach service | Social Mobilization | Supply Chain |
| Uganda | <0.001 | <0.001 | <0.001 | <0.001 | <0.001 | <0.001 |
| Benin | <0.001 | <0.001 | <0.001 | 0.144 | <0.001 | <0.001 |
| Ghana | <0.001 | <0.001 | <0.001 | <0.001 | <0.001 | <0.001 |
| Zambia | <0.001 | <0.001 | <0.001 | <0.001 | <0.001 | <0.001 |
| Moldova | <0.001 | <0.001 | <0.001 | --- | <0.001 | <0.001 |
| Honduras | <0.001 | <0.001 | <0.001 | <0.001 | <0.001 | <0.001 |

Table S32. Statistical testing results for differences in the cost shares estimated for the sites with lowest and highest efficiency level, across budget category.

| Country | P value of two-tailed t-test for estimated differences of cost share by budget category | | | | | |
| --- | --- | --- | --- | --- | --- | --- |
|  | Labor | Vaccine | Cold Chain | Vehicle | Infrastructure | Per Diem |
| Uganda | <0.001 | <0.001 | <0.001 | <0.001 | <0.001 | <0.001 |
| Benin | <0.001 | <0.001 | 0.782 | <0.001 | <0.001 | <0.001 |
| Ghana | <0.001 | <0.001 | <0.001 | <0.001 | <0.001 | <0.001 |
| Zambia | <0.001 | <0.001 | <0.001 | <0.001 | <0.001 | <0.001 |
| Moldova | <0.001 | <0.001 | <0.001 | <0.001 | <0.001 | <0.001 |
| Honduras | <0.001 | <0.001 | <0.001 | --- | <0.001 | <0.001 |

Table S33. Statistical testing results for differences in the cost shares estimated for the sites with lowest and highest efficiency level, across programmatic activity.

| Country | P value of two-tailed t-test for estimated differences of cost share by programmatic activity | | | | | |
| --- | --- | --- | --- | --- | --- | --- |
|  | Facility-based service | Surveillance | Program Management | Outreach service | Social Mobilization | Supply Chain |
| Uganda | <0.001 | <0.001 | <0.001 | <0.001 | <0.001 | 0.050 |
| Benin | <0.001 | <0.001 | <0.001 | <0.001 | <0.001 | <0.001 |
| Ghana | <0.001 | <0.001 | <0.001 | <0.001 | <0.001 | <0.001 |
| Zambia | <0.001 | <0.001 | <0.001 | <0.001 | <0.001 | <0.001 |
| Moldova | <0.001 | <0.001 | <0.001 | --- | <0.001 | <0.001 |
| Honduras | <0.001 | <0.001 | <0.001 | <0.001 | <0.001 | <0.001 |
